# Supplementary material for: Expression quantitative trait loci-derived scores and white matter microstructure in UK Biobank: a novel approach to integrating genetics and neuroimaging
Source: Transl Psychiatry. 2020 Feb 4;10:55. doi: 10.1038/s41398-020-0724-y (PMC7026054; doi:10.1038/s41398-020-0724-y)
Supplement: Supplementary file 1 — Supplementary materials [file 41398_2020_724_MOESM1_ESM.docx]

**Supplementary materials**

1. **eQTL score computation process (figure S1)**
2. **Individual white matter tracts and tract category to which they belong (table S1)**
3. **R code for linear models (created using R Markdown)**

eQTL analysis

Miruna Barbu

## Data analysis - unilateral white matter tracts, global, and regional measures

# Read in dataset
#FA_short = readRDS("/path-to-file/file.rds")

# Run linear model for unilateral tracts
n <- 15 ## Change according to whatever number of scores are calculated

# Run a linear model including each score separately in relation to global FA; include covariates first in model, and then eQTL score

my_lms_no_quadr <- lapply(2:n, function(x) lm(FA_short$global_FA ~ FA_short$MRI_age.calculated+I(FA_short$MRI_age.calculated^2)+FA_short$sex+FA_short$pc1+FA_short$pc2+FA_short$pc3+FA_short$pc4+ FA_short$pc5+FA_short$pc6+FA_short$pc7+FA_short$pc8+FA_short$pc9+FA_short$pc10+FA_short$pc11+FA_short$pc12+FA_short$pc13+FA_short$pc14+FA_short$pc15+ FA_short$genotyping.array+FA_short$pos.x+FA_short$pos.y+FA_short$pos.z+FA_short$MRI_site+ FA_short[,x]))

# Extract summary from each model in a list
summaries <- lapply(my_lms_no_quadr, summary)

# Extract coefficients table for each eQTL score in a list (in this case, it is the 25th line)
stats = lapply(summaries, function(x) x$coefficients[c(25),])
# Name the stats list according to each eQTL score in original dataset
names(stats) = colnames(FA_short)[2:15] ## Change according to scores
# Turn into a dataframe
stats_dataframe = data.frame(stats)
# Extract R2 and adjusted R2 for each model
rsq = sapply(summaries, function(x) c(r_sq = x$r.squared, adj_r_sq = x$adj.r.squared))
# Name the R2 list according to each eQTL score in original dataset
colnames(rsq) = colnames(FA_short)[2:15] ## Change according to scores
# put coefficients and R2 together in a dataset, naming the dataset according to tract investigated
FA_global = rbind(stats_dataframe,rsq)
# Transpose
FA_global = t(FA_global)
FA_global = data.frame(FA_global)

print(FA_global)

## Estimate Std..Error t.value Pr...t.. r_sq
## X14794_1 -0.03710861 0.007892813 -4.701569 2.605478e-06 0.09710308
## X16411_1 -0.02267562 0.007896269 -2.871688 4.088783e-03 0.09624023
## X16970_1 -0.04024645 0.007892880 -5.099083 3.456073e-07 0.09734536
## X17549_1 -0.02423319 0.007897119 -3.068612 2.154527e-03 0.09631312
## X10929_0 0.37073357 0.096298017 3.849857 1.186947e-04 0.09664980
## X2040_1 -0.10626922 0.028722802 -3.699821 2.165496e-04 0.09657926
## X2406_1 -0.16386563 0.042176139 -3.885269 1.026713e-04 0.09666686
## X2943_0 0.15967411 0.137795342 1.158777 2.465661e-01 0.09580975
## X2943_1 -0.21908305 0.049652765 -4.412303 1.030149e-05 0.09693906
## X4537_1 0.22222069 0.070059600 3.171881 1.517710e-03 0.09635328
## X4619_1 -0.01876633 0.085763474 -0.218815 8.267972e-01 0.09572897
## X5582_0 -0.14887362 0.122100620 -1.219270 2.227616e-01 0.09581873
## X6806_0 -0.68569122 0.130077801 -5.271393 1.373349e-07 0.09745644
## X710_1 -0.08041791 0.021438769 -3.751051 1.767809e-04 0.09660304
## adj_r_sq
## X14794_1 0.09560791
## X16411_1 0.09474362
## X16970_1 0.09585059
## X17549_1 0.09481664
## X10929_0 0.09515388
## X2040_1 0.09508323
## X2406_1 0.09517096
## X2943_0 0.09431244
## X2943_1 0.09544362
## X4537_1 0.09485687
## X4619_1 0.09423152
## X5582_0 0.09432143
## X6806_0 0.09596185
## X710_1 0.09510704

# Write table containing results
# write.table(FA_global,"/path-to-file/FA_global.txt", quote = F)
knitr::opts_chunk$set(echo = TRUE)

## Data analysis - bilateral white matter tracts

# Read in dataset

#FA_long = readRDS("/path-to-file/file.rds")
library(nlme)

# Select all eQTL scores in dataset and include in object
WM_list <- colnames(FA_long)[2:15]
no_WM_list <- length(WM_list) # assign length of columns

# create a named list to hold the fitted models
fitlist <- as.list(1:no_WM_list)
names(fitlist) <- WM_list # assign eQTL scores as names to list

# Run a linear model loop for each white matter tract
for(i in WM_list){
 fml <- as.formula( paste("FA.wm.acoustic_radiation", "~", paste(c("sex","I(MRI_age.calculated^2)","MRI_age.calculated","pc1","pc2","pc3","pc4","pc5","pc6","pc7","pc8","pc9","pc10","pc11","pc12","pc13","pc14","pc15","genotyping.array","pos.x", "pos.y","pos.z","MRI_site","hemi",i), collapse="+") ) )

fitlist[[i]] <- lme(fml, random=~1|f.eid,na.action=na.exclude,control=lmeControl(opt = "optim"), data=FA_long)
}

# Extract summary from each model in a list
summaries_ar <- lapply(fitlist, summary)
# Extract coefficients table for each eQTL score in a list (in this case, it is the 26th line)
stats_ar = lapply(summaries_ar, function(x) x$tTable[c(26),])
# Name the stats list according to each eQTL score in original dataset
names(stats_ar) = colnames(FA_long)[2:15]
# Turn into dataframe
stats_dataframe_ar = data.frame(stats_ar)
# Transpose
stats_dataframe_ar = t(stats_dataframe_ar)

print(stats_dataframe_ar)

## Value Std.Error DF t-value p-value
## X14794_1 -0.014526420 0.006934398 14493 -2.0948352 3.620296e-02
## X16411_1 -0.002867261 0.006935130 14493 -0.4134401 6.792903e-01
## X16970_1 -0.032609314 0.006931150 14493 -4.7047481 2.565272e-06
## X17549_1 -0.007131708 0.006935944 14493 -1.0282245 3.038614e-01
## X10929_0 0.153309002 0.084586648 14493 1.8124492 6.993750e-02
## X2040_1 -0.055011121 0.025227390 14493 -2.1806108 2.922824e-02
## X2406_1 -0.145210581 0.037031773 14493 -3.9212430 8.849903e-05
## X2943_0 0.122759957 0.120990405 14493 1.0146255 3.103013e-01
## X2943_1 -0.181800620 0.043600001 14493 -4.1697389 3.067221e-05
## X4537_1 0.044163220 0.061535016 14493 0.7176925 4.729585e-01
## X4619_1 0.042266845 0.075302614 14493 0.5612932 5.746064e-01
## X5582_0 -0.110125091 0.107210182 14493 -1.0271887 3.043487e-01
## X6806_0 -0.385959442 0.114277340 14493 -3.3773926 7.336899e-04
## X710_1 -0.022695943 0.018832176 14493 -1.2051684 2.281580e-01

# Install package to extract marginal and conditional R2 from model
library(MuMIn)

# Extract marginal and conditional R2 from each list item
r_squared_ar = lapply(fitlist, function(x) r.squaredGLMM(x))

## Warning: 'r.squaredGLMM' now calculates a revised statistic. See the help
## page.

# Turn this to list format to then turn into dataframe
r_squared_output_ar = lapply(r_squared_ar, function(x) c(r2m = x[1], r2c = x[2]))
data_r = data.frame(r_squared_output_ar) # Turn into dataframe
data_r = t(data_r) # Transpose

print(data_r)

## r2m r2c
## X14794_1 0.07912247 0.5620881
## X16411_1 0.07904833 0.5621498
## X16970_1 0.07947079 0.5618462
## X17549_1 0.07903623 0.5621237
## X10929_0 0.08230257 0.5636257
## X2040_1 0.07941558 0.5622191
## X2406_1 0.08158552 0.5630076
## X2943_0 0.08119967 0.5631530
## X2943_1 0.08296838 0.5636196
## X4537_1 0.07939716 0.5623078
## X4619_1 0.07933646 0.5622835
## X5582_0 0.08034324 0.5627452
## X6806_0 0.09675071 0.5703128
## X710_1 0.07910257 0.5621462

# Write tables containing results
# write.table(stats_dataframe,"/path-to-file/FA_acoustic_radiation.txt", quote = F)
# write.table(data_r,"/path-to-file/FA_acoustic_radiation_r2.txt", quote = F)

knitr::opts_chunk$set(echo = TRUE)

1. **eQTL quadratic term inclusion in linear models (tables S2 – S3; figures S2 – S3)**

Quadratic terms for each eQTL score were included in separate linear regression models in order to observe whether there are non-linear relationships between the eQTL scores and brain connectivity measures. Global and regional tracts were regressed on each score and its quadratic term in separate linear models (N = 10,857 eQTL scores across the two discovery datasets, Gusev et al. and Westra et al.), with the following covariates included: sex, age, age^2^, 15 genetic principal components, genotype array, 3 MRI head position covariates, and MRI site. FDR-corrected p-value and effect size were extracted for each quadratic term. Additionally, increase in variance explained by adding the quadratic term was explored by subtracting the linear model R^2^ from the quadratic-inclusive model R^2^. Tables S2 and S3 and figures S2 and S3 provide a summary of the results from this analysis.

1. **Reliability of findings across the two eQTL discovery datasets for the 8 top genes identified (Tables S4 – S12).**

For the current manuscript’s main analysis, overlapping eQTL scores (i.e. eQTL scores that regulated the expression of the same gene) between the two discovery datasets were investigated for significance, and the score with the most significant association within the discovery dataset was selected. The eQTL scores that were significantly associated with both FA and MD across numerous tracts in the main manuscript were later compared with any overlapping eQTL scores for those same genes in order to observe whether findings replicate across independent discovery datasets.

Four of the eight eQTL scores were present in both discovery datasets: *DCAKD* (both *cis* scores); *SLC35A4* (one *cis* score in Gusev et al.; one *cis* and one *trans* score in Westra et al.); *UBE3C* (one *cis* score in Gusev et al.; two *cis* scores in Westra et al.); and *PLEKHM1* (one *cis* score in Gusev et al. and one *cis* score in Westra et al., which was for *PLEKHM1P1*, a pseudogene for *PLEKHM1*). These results are presented below in tables for each score and for each tract that was significantly associated with the score in the main analysis.

In summary, *cis* eQTL scores for *DCAKD* and *SLC35A4* from both databases explained a similar proportion of variance in significantly-associated white matter tracts, while the *trans* eQTL score for *SLC35A4* did not, indicating differences between *cis* and *trans* eQTL. We note that eQTL scores for *PLEKHM1* and *UBE3C* selected from the two databases demonstrated greater differences in proportion of variance explained. The eQTL scores for *PLEKHM1* and *UBE3C* included in the main manuscript were selected based on the most significant association between SNPs and expression of the genes; the scores present in the main manuscript explain a greater amount of variance compared with the second database’s scores, strengthening the rationale for selecting the strongest association between the two databases.

More specifically, the eQTL scores for *DCAKD* explained a similar proportion of variance in global FA (0.082% and 0.132%), global MD (0.166% and 0.146%), thalamic radiations (FA: 0.101% and 0.159%; MD: 0.109% and 0.094%), forceps minor (FA: 0.150% and 0.221%; MD: 0.232% and 0.185%), and association fibres (MD: 0.145% and 0.139%). The two scores explained little or no variance in FA-measured superior longitudinal fasciculus (0.001% and 0%) and anterior thalamic radiations (0.001% and 0%), and in MD-measured acoustic radiation (0.% and 0.002%), uncinate fasciculus (0% and 0.001%), cingulate gyrus (0% and 0.001%), inferior longitudinal fasciculus (0% and 0.001%), anterior thalamic radiations (0% and 0%), inferior fronto-occipital fasciculus (0% and 0.001%), and superior longitudinal fasciculus (0% and 0%).

The eQTL scores (*cis* only) for *SLC35A4* explained a similar proportion of variance in global FA (0.156% and 0.115%), global MD (0.088% and 0.076%), forceps minor (FA: 0.025% and 0.001%; MD: 0.181% and 0.143%), FA association fibres (0.114% and 0.074%) and FA projection fibres (0.201% and 0.143%). Interestingly, the *trans* eQTL for *SLC35A4* explains little or no variance in these tracts (FA: global FA=0.002%, forceps minor=0%, association fibres=0.006%, projection fibres=0%; MD: global MD and forceps minor=0%), indicating that eQTL scores in *trans* may act through different regulatory processes in the context of white matter microstructure.

The eQTL scores for *PLEKHM1* do not explain a similar proportion of variance in any of the tracts found to be significant for the score in the main manuscript. This may be due to the second discovery dataset’s gene being a pseudogene, which is a segment of DNA that is related to the real gene, but is an imperfect copy.

Finally, the eQTL scores for *UBE3C* did not explain a similar amount of variance for tracts either in FA (forceps minor: 0.052%; 0.141%; 0.002%) or MD (forceps minor: 0.039%; 0.104%; 0.004% and inferior fronto-occipital fasciculus: 0%; 0.22%; 0.148%).

1. **Results for 8 scores associated with both FA and MD (N = 8; tables S13 – S20).**
2. **Results for scores associated with FA (N = 17; table S21; figure S4) and MD (N = 16; table S22; figure S5) white matter tracts separately**
3. **Brief gene look-up for genes whose expression was associated with FA (N = 17; table S23) and MD (N = 16; table S24) separately**
4. **Allen Brain Atlas search of 8 top genes (Table S25; Figures S6 – S8).**
5. **P-values and effect size for each SNP in association with gene expression as taken from GENOSCORES; these values were obtained in the two discovery datasets used in the current study (Gusev et al., 2016; Westra et al., 2013; Table S26).**
6. **GWAS quality check and parameters**

In order to determine whether any score SNPs were previously associated with global and tract category measures of interest (i.e. tract categories & global measures found to be significantly associated with the 8 eQTL scores), we ran 8 GWAS locally (the 3 tract categories: association fibres, thalamic radiations, and projection fibres for both FA and MD, and global measures for FA and MD). We used BGENIE (1) to conduct the association analysis and excluded related participants (up to the third degree using the KING toolset (2)), as well as those who also participated in Generation Scotland and PGC MDD GWAS. Only variants with a minor allele frequency (MAF) > 0.001 (0.1%), SNP information score (quality of imputation) > 0.1, and Hardy-Weinberg equilibrium (HWE) p-value >= 1e-6 were examined. Sex, age, the first 8 principal components, genotyping array, and three head position coordinates were fitted as covariates in the analysis.

The output summary statistics files contain information with regards to the chromosome, SNP ID, p-value and effect size of association with each phenotype. We looked up the SNPs significantly associated with the tracts of interest, and noted the effect size and p-value for each.

1. **P-values and effect size of each SNP for each individual white matter tract results (Elliott et al., 2018) and global and regional results (run locally); figure produced locally (figure S9)**

**Supplementary material references**

1. Phillips, A. R., Suttangkakul, A., & Vierstra, R. D. (2008). The ATG12-conjugating enzyme ATG10 is essential for autophagic vesicle formation in Arabidopsis thaliana. *Genetics*, *178*(3), 1339-1353.
2. Sharma, S., Wongpalee, S. P., Vashisht, A., Wohlschlegel, J. A., & Black, D. L. (2014). Stem–loop 4 of U1 snRNA is essential for splicing and interacts with the U2 snRNP-specific SF3A1 protein during spliceosome assembly. *Genes & development*, *28*(22), 2518-2531.
3. Bansbach, C. E., Bétous, R., Lovejoy, C. A., Glick, G. G., & Cortez, D. (2009). The annealing helicase SMARCAL1 maintains genome integrity at stalled replication forks. *Genes & development*, *23*(20), 2405-2414.
4. Christopher, L., Napolioni, V., Khan, R. R., Han, S. S., Greicius, M. D., & Alzheimer's Disease Neuroimaging Initiative. (2017). A variant in PPP4R3A protects against alzheimer‐related metabolic decline. *Annals of neurology*, *82*(6), 900-911.
5. Wright, S. D., Ramos, R. A., Tobias, P. S., Ulevitch, R. J., & Mathison, J. C. (1990). CD14, a receptor for complexes of lipopolysaccharide (LPS) and LPS binding protein. *Science*, *249*(4975), 1431-1433.
6. Steet, R., & Kornfeld, S. (2006). COG-7-deficient human fibroblasts exhibit altered recycling of Golgi proteins. *Molecular biology of the cell*, *17*(5), 2312-2321.
7. Morava, E., Zeevaert, R., Korsch, E., Huijben, K., Wopereis, S., Matthijs, G. et al. (2007). A common mutation in the COG7 gene with a consistent phenotype including microcephaly, adducted thumbs, growth retardation, VSD and episodes of hyperthermia. *European Journal of Human Genetics*, *15*(6), 638.
8. Qin, Z., Wang, Y., Tang, J., Zhang, L., Li, R., Xue, J. et al. (2018). High LINC01605 expression predicts poor prognosis and promotes tumor progression via up-regulation of MMP9 in bladder cancer. *Bioscience reports*, *38*(5), BSR20180562.
9. Massé, K. L., Collins, R., Bhamra, S., Seville, R. A., & Jones, E. (2007). Anxa4 genes are expressed in distinct organ systems in xenopus laevis and tropicalis but are functionally conserved. *Organogenesis*, *3*(2), 83-92.
10. Ergin, V., Erdogan, M., & Menevse, A. (2015). Regulation of shootin1 gene expression involves ngf-induced alternative splicing during neuronal differentiation of PC12 cells. *Scientific reports*, *5*, 17931.
11. Yeo, S. Y., Ha, S. Y., Yu, E. J., Lee, K. W., Kim, J. H., & Kim, S. H. (2014). ZNF282 (Zinc finger protein 282), a novel E2F1 co-activator, promotes esophageal squamous cell carcinoma. *Oncotarget*, *5*(23), 12260.
12. Edqvist, P. H. D., Huvila, J., Forsström, B., Talve, L., Carpén, O., Salvesen, H. B. et al. (2015). Loss of ASRGL1 expression is an independent biomarker for disease-specific survival in endometrioid endometrial carcinoma. *Gynecologic oncology*, *137*(3), 529-537.
13. Bhattacharya, M. R., Geisler, S., Pittman, S. K., Doan, R. A., Weihl, C. C., Milbrandt, J. et al. (2016). TMEM184b promotes axon degeneration and neuromuscular junction maintenance. *Journal of Neuroscience*, *36*(17), 4681-4689.
14. Wang, L., Wildt, K. F., Castro, E., Xiong, Y., Feigenbaum, L., Tessarollo, L. et al. (2008). The zinc finger transcription factor Zbtb7b represses CD8-lineage gene expression in peripheral CD4+ T cells. *Immunity*, *29*(6), 876-887.
15. Nesbit, M. A., Hannan, F. M., Howles, S. A., Reed, A. A., Cranston, T., Thakker, C. E. et al. (2013). Mutations in AP2S1 cause familial hypocalciuric hypercalcemia type 3. *Nature genetics*, *45*(1), 93.
16. Spiegel, R., Shaag, A., Shalev, S., & Elpeleg, O. (2016). Homozygous mutation in the APOA1BP is associated with a lethal infantile leukoencephalopathy. *Neurogenetics*, *17*(3), 187-190.
17. Zhu, M., Yan, C., Ren, C., Huang, X., Zhu, X., Gu, H. et al. (2017). Exome array analysis identifies variants in SPOCD1 and BTN3A2 that affect risk for gastric cancer. *Gastroenterology*, *152*(8), 2011-2021.
18. Evans, D. R., & Guy, H. I. (2004). Mammalian pyrimidine biosynthesis: fresh insights into an ancient pathway. *Journal of Biological Chemistry*, *279*(32), 33035-33038.
19. Maxson, J. E., Gotlib, J., Pollyea, D. A., Fleischman, A. G., Agarwal, A., Eide, C. A. et al. (2013). Oncogenic CSF3R mutations in chronic neutrophilic leukemia and atypical CML. *New England Journal of Medicine*, *368*(19), 1781-1790.
20. Rhee, H. W., Zou, P., Udeshi, N. D., Martell, J. D., Mootha, V. K., Carr, S. A. et al. (2013). Proteomic mapping of mitochondria in living cells via spatially restricted enzymatic tagging. *Science*, *339*(6125), 1328-1331.
21. Zollino, M., Orteschi, D., Murdolo, M., Lattante, S., Battaglia, D., Stefanini, C. et al. (2012). Mutations in KANSL1 cause the 17q21. 31 microdeletion syndrome phenotype. *Nature genetics*, *44*(6), 636.
22. Zhernakova, A., Festen, E. M., Franke, L., Trynka, G., van Diemen, C. C., Monsuur, A. J. et al. (2008). Genetic analysis of innate immunity in Crohn's disease and ulcerative colitis identifies two susceptibility loci harboring CARD9 and IL18RAP. *The American Journal of Human Genetics*, *82*(5), 1202-1210.
23. Gertow, K., Sennblad, B., Strawbridge, R. J., Öhrvik, J., Zabaneh, D., Shah, S. et al. (2012). Identification of the BCAR1-CFDP1-TMEM170A locus as a determinant of carotid intima-media thickness and coronary artery disease risk. *Circulation: Cardiovascular Genetics*, *5*(6), 656-665.
24. Zhu, J. H., Chen, R., Yi, W., Cantin, G. T., Fearns, C., Yang, Y. et al. (2008). Protein tyrosine phosphatase PTPN13 negatively regulates Her2/ErbB2 malignant signaling. *Oncogene*, *27*(18), 2525.
25. Wills, Z., Bateman, J., Korey, C. A., Comer, A., & Van Vactor, D. (1999). The tyrosine kinase Abl and its substrate enabled collaborate with the receptor phosphatase Dlar to control motor axon guidance. *Neuron*, *22*(2), 301-312.
26. Niwa, T., Saito, H., Imajoh‐ohmi, S., Kaminishi, M., Seto, Y., Miki, Y. et al. (2009). BRCA2 interacts with the cytoskeletal linker protein plectin to form a complex controlling centrosome localization. *Cancer science*, *100*(11), 2115-2125.
27. Bycroft, C., Freeman, C., Petkova, D., Band, G., Elliott, L. T., Sharp, K. et al. (2018). The UK Biobank resource with deep phenotyping and genomic data. Nature, 562(7726), 203.
28. Manichaikul, A., Mychaleckyj, J. C., Rich, S. S., Daly, K., Sale, M., & Chen, W. M. (2010). Robust relationship inference in genome-wide association studies. Bioinformatics, 26(22), 2867-2873.
29. Shen, E. H., Overly, C. C., & Jones, A. R. (2012). The Allen Human Brain Atlas: comprehensive gene expression mapping of the human brain. *Trends in neurosciences*, *35*(12), 711-714.

| **Fractional anisotropy and mean diffusivity** |
| --- |
| *Association fibres* |
| Inferior fronto-occipital fasciculus |
| Uncinate fasciculus |
| Parahippocampal cingulum |
| Cingulate gyrus |
| Superior longitudinal fasciculus |
| Inferior longitudinal fasciculus |
| *Thalamic radiations* |
| Anterior thalamic radiation |
| Posterior thalamic radiation |
| Superior thalamic radiation |
| *Projection fibres* |
| Acoustic radiation |
| Medial lemniscus |
| Forceps major* |
| Forceps minor* |
| Middle cerebellar peduncle* |
| Corticospinal tract |
| *Global FA & global MD* |
| **Table S1.** White matter tracts, global white matter and tract categories for FA and MD.  * indicates unilateral regions. |

| **White matter tract** | **FDR-corrected scores (N)** | **More than 0.05% R^2^ variance explained** |
| --- | --- | --- |
| Global | None | 39 |
| Association fibres | None | 37 |
| Thalamic radiations | None | 44 |
| Projection fibres | None | 40 |

**Table S2**. FA global and regional associations with quadratic terms of eQTL scores. None of the quadratic terms survived FDR correction; a low number (37 – 44 across general and regional measures) of eQTL scores increased variance explained by more than 0.05% in the global and tract category measures above.

| **White matter tract** | **FDR-corrected scores (N)** | **More than 0.05% R^2^ variance explained** |
| --- | --- | --- |
| Global | None | 22 |
| Association fibres | None | 28 |
| Thalamic radiations | None | 10 |
| Projection fibres | None | 33 |

**Table S3**. MD global and regional associations with quadratic terms of eQTL scores. None of the quadratic terms survived FDR correction; a low number (10 – 33 across general and regional measures) of eQTL scores increased variance explained by more than 0.05% in the global and tract category measures above.

| **White matter tract** | **Abbreviation** |
| --- | --- |
| Global FA | gFA |
| Association fibres | AF |
| Thalamic radiations | TR |
| Projection fibres | PF |
| Superior longitudinal fasciculus | SLF |
| Anterior thalamic radiations | ATR |
| Forceps minor | F-min |
| Corticospinal tract | CST |
| Acoustic radiation | AR |
| Inferior longitudinal fasciculus | ILF |
| Uncinate fasciculus | UF |
| Cingulate gyrus | CGC |
| Inferior fronto-occipital fasciculus | IFOF |

**Table S4.** White matter tracts included in tables 2 – 9 below.

| **eQTL score** | **White matter tract** | **Study from which score is calculated** | **Effect size** | **SD** | **t value** | **p value** | **R2 (%)** |
| --- | --- | --- | --- | --- | --- | --- | --- |
| DCAKD_cis | gFA | Westra et al. | -0.080 | 0.021 | -3.751 | < 0.001 | 0.082 |
| *****DCAKD_cis | gFA | Gusev et al. | -0.037 | 0.008 | -4.702 | < 0.001 | 0.132 |
| DCAKD_cis | TR | Westra et al. | -0.089 | 0.022 | -4.108 | < 0.001 | 0.101 |
| *****DCAKD_cis | TR | Gusev et al. | -0.041 | 0.008 | -5.083 | < 0.001 | 0.159 |
| DCAKD_cis | SLF | Gusev et al. | -0.086 | 0.021 | -4.147 | < 0.001 | 0.001 |
| *****DCAKD_cis | SLF | Westra et al. | -0.039 | 0.008 | -5.074 | < 0.001 | 0 |
| DCAKD_cis | ATR | Gusev et al. | -0.095 | 0.021 | -4.612 | < 0.001 | 0.001 |
| *****DCAKD_cis | ATR | Westra et al. | -0.043 | 0.008 | -5.710 | < 0.001 | 0 |
| DCAKD_cis | F-min | Westra et al. | -0.107 | 0.021 | -5.021 | 5.2 × 10^-7^ | 0.150 |
| *****DCAKD_cis | F-min | Gusev et al. | -0.047 | 0.008 | -6.068 | 1.3 × 10^-9^ | 0.221 |

**Table S5**. Comparison between eQTL scores for *DCAKD* extracted from both discovery datasets in their association with white matter tracts (FA); * = eQTL score based on significant associations in the discovery dataset for the main analysis included in manuscript.

| **eQTL score** | **White matter tract** | **Study from which score is calculated** | **Effect size** | **SD** | **t value** | **p value** | **R2 (%)** |
| --- | --- | --- | --- | --- | --- | --- | --- |
| *****SLC35A4_cis | gFA | Gusev et al. | -0.040 | 0.008 | -5.099 | < 0.001 | 0.156 |
| SLC35A4_trans | gFA | Westra et al. | 0.160 | 0.138 | 1.159 | 0.247 | 0.002 |
| SLC35A4_cis | gFA | Westra et al. | -0.219 | 0.050 | -4.412 | < 0.001 | 0.115 |
| *****SLC35A4_cis | AF | Gusev et al. | -0.035 | 0.008 | -4.403 | < 0.001 | 0.114 |
| SLC35A4_trans | AF | Westra et al. | 0.193 | 0.137 | 1.402 | 0.161 | 0.006 |
| SLC35A4_cis | AF | Westra et al. | -0.178 | 0.050 | -3.589 | < 0.001 | 0.074 |
| *****SLC35A4_cis | PF | Gusev et al. | -0.045 | 0.008 | -5.760 | < 0.001 | 0.201 |
| SLC35A4_trans | PF | Westra et al. | 0.098 | 0.137 | 0.714 | 0.476 | 0 |
| SLC35A4_cis | PF | Westra et al. | -0.242 | 0.050 | -4.889 | < 0.001 | 0.143 |
| *****SLC35A4_cis | CST | Gusev et al. | -0.033 | 0.007 | -4.395 | < 0.001 | 0 |
| SLC35A4_trans | CST | Westra et al. | -0.022 | 0.129 | -0.171 | 0.864 | 0.002 |
| SLC35A4_cis | CST | Westra et al. | -0.164 | 0.047 | -3.526 | < 0.001 | 0.066 |
| *****SLC35A4_cis | AR | Gusev et al. | -0.033 | 0.007 | -4.705 | < 0.001 | 0 |
| SLC35A4_trans | AR | Westra et al. | 0.123 | 0.121 | 1.015 | 0.310 | 0.103 |
| SLC35A4_cis | AR | Westra et al. | -0.182 | 0.044 | -4.170 | < 0.001 | 0.149 |
| *****SLC35A4_cis | ILF | Gusev et al. | -0.034 | 0.008 | -4.387 | < 0.001 | 0 |
| SLC35A4_trans | ILF | Westra et al. | 0.143 | 0.133 | 1.070 | 0.285 | 0.043 |
| SLC35A4_cis | ILF | Westra et al. | -0.207 | 0.048 | -4.316 | < 0.001 | 0.066 |
| *****SLC35A4_cis | SLF | Gusev et al. | -0.037 | 0.008 | -4.787 | < 0.001 | 0 |
| SLC35A4_trans | SLF | Westra et al. | 0.165 | 0.134 | 1.234 | 0.217 | 0.051 |
| SLC35A4_cis | SLF | Westra et al. | -0.193 | 0.048 | -4.000 | < 0.001 | 0.053 |
| *****SLC35A4_cis | F-min | Gusev et al. | -0.018 | 0.008 | -2.179 | 0.029 | 0.025 |
| SLC35A4_trans | F-min | Westra et al. | 0.036 | 0.143 | 0.251 | 0.802 | 0 |
| SLC35A4_cis | F-min | Westra et al. | -0.056 | 0.052 | -1.078 | 0.281 | 0.001 |

**Table S6**. Comparison between eQTL scores for *SLC35A4* extracted from both discovery datasets in their association with white matter tracts (FA); * = eQTL score based on significant associations in the discovery dataset for the main analysis included in manuscript.

| **eQTL score** | **White matter tract** | **Study from which score is calculated** | **Effect size** | **SD** | **t value** | **p value** | **R2 (%)** |
| --- | --- | --- | --- | --- | --- | --- | --- |
| PLEKHM1P1_cis | F-min | Westra et al. | -0.076 | 0.085 | -0.892 | 3.7 × 10^-1^ | 0 |
| *****PLEKHM1_cis-x | F-min | Gusev et al. | -0.035 | 0.008 | -4.502 | 6.8 × 10^-6^ | 0.119 |

**Table S7.** Comparison between eQTL scores for *PLEKHM1* extracted from both discovery datasets in their association with white matter tracts (FA); * = eQTL score based on significant associations in the discovery dataset for the main analysis included in manuscript.

| **eQTL score** | **White matter tract** | **Study from which score is calculated** | **Effect size** | **SD** | **t value** | **p value** | **R2 (%)** |
| --- | --- | --- | --- | --- | --- | --- | --- |
| UBE3C_cis | F-min | Gusev et al. | -0.024 | 0.008 | -3.064 | 2.2 × 10^-3^ | 0.052 |
| *****UBE3C_cis | F-min | Westra et al. | -0.139 | 0.028 | -4.883 | 1.1 × 10^-6^ | 0.141 |
| UBE3C_cis | F-min | Westra et al. | -0.137 | 0.121 | -1.131 | 2.6 × 10^-1^ | 0.002 |

**Table S8.** Comparison between eQTL scores for *UBE3C* extracted from both discovery datasets in their association with white matter tracts (FA); * = eQTL score based on significant associations in the discovery dataset for the main analysis included in manuscript.

| **eQTL score** | **White matter tract** | **Study from which score is calculated** | **Effect size** | **SD** | **t value** | **p value** | **R2 (%)** |
| --- | --- | --- | --- | --- | --- | --- | --- |
| DCAKD_cis | gMD | Gusev et al. | 0.042 | 0.007 | 5.562 | < 0.001 | 0.166 |
| *****DCAKD_cis | gMD | Westra et al. | 0.106 | 0.020 | 5.235 | < 0.001 | 0.146 |
| DCAKD_cis | TR | Gusev et al. | 0.034 | 0.007 | 4.778 | < 0.001 | 0.109 |
| *****DCAKD_cis | TR | Westra et al. | 0.086 | 0.019 | 4.441 | < 0.001 | 0.094 |
| DCAKD_cis | AF | Gusev et al. | 0.039 | 0.008 | 5.112 | < 0.001 | 0.145 |
| *****DCAKD_cis | AF | Westra et al. | 0.104 | 0.021 | 5.012 | < 0.001 | 0.139 |
| DCAKD_cis | AR | Gusev et al. | 0.030 | 0.007 | 4.291 | < 0.001 | 0 |
| *****DCAKD_cis | AR | Westra et al. | 0.074 | 0.019 | 3.909 | < 0.001 | 0.002 |
| DCAKD_cis | UF | Gusev et al. | 0.032 | 0.007 | 4.753 | < 0.001 | 0 |
| *****DCAKD_cis | UF | Westra et al. | 0.077 | 0.018 | 4.166 | < 0.001 | 0.001 |
| DCAKD_cis | CGC | Gusev et al. | 0.035 | 0.007 | 4.834 | < 0.001 | 0 |
| *****DCAKD_cis | CGC | Westra et al. | 0.090 | 0.020 | 4.495 | < 0.001 | 0.001 |
| DCAKD_cis | ILF | Gusev et al. | 0.038 | 0.007 | 5.285 | < 0.001 | 0 |
| *****DCAKD_cis | ILF | Westra et al. | 0.097 | 0.020 | 4.908 | < 0.001 | 0.001 |
| DCAKD_cis | ATR | Gusev et al. | 0.041 | 0.007 | 6.016 | < 0.001 | 0 |
| *****DCAKD_cis | ATR | Westra et al. | 0.105 | 0.019 | 5.600 | < 0.001 | 0.000 |
| DCAKD_cis | IFOF | Gusev et al. | 0.042 | 0.007 | 5.588 | < 0.001 | 0 |
| *****DCAKD_cis | IFOF | Westra et al. | 0.103 | 0.020 | 5.082 | < 0.001 | 0.001 |
| DCAKD_cis | SLF | Gusev et al. | 0.042 | 0.008 | 5.608 | < 0.001 | 0 |
| *****DCAKD_cis | SLF | Westra et al. | 0.110 | 0.020 | 5.351 | < 0.001 | 0.000 |
| DCAKD_cis | F-min | Gusev et al. | 0.049 | 0.008 | 6.432 | < 0.001 | 0.232 |
| *****DCAKD_cis | F-min | Westra et al. | 0.118 | 0.021 | 5.750 | < 0.001 | 0.185 |

**Table S9.** Comparison between eQTL scores for *DCAKD* extracted from both discovery datasets in their association with white matter tracts (MD); * = eQTL score based on significant associations in the discovery dataset for the main analysis included in manuscript.

| **eQTL score** | **White matter tract** | **Study from which score is calculated** | **Effect size** | **SD** | **t value** | **p value** | **R2 (%)** |
| --- | --- | --- | --- | --- | --- | --- | --- |
| *****SLC35A4_cis | gMD | Gusev et al. | 0.031 | 0.007 | 4.110 | < 0.001 | 0.088 |
| SLC35A4_trans | gMD | Westra et al. | -0.122 | 0.131 | -0.936 | 0.349 | 0 |
| SLC35A4_cis | gMD | Westra et al. | 0.180 | 0.047 | 3.837 | < 0.001 | 0.076 |
| *****SLC35A4_cis | ILF | Gusev et al. | 0.036 | 0.007 | 4.976 | < 0.001 | 0 |
| SLC35A4_trans | ILF | Westra et al. | -0.180 | 0.127 | -1.414 | 0.157 | 0.062 |
| SLC35A4_cis | ILF | Westra et al. | 0.211 | 0.046 | 4.614 | < 0.001 | 0.070 |
| *****SLC35A4_cis | F-min | Gusev et al. | 0.043 | 0.008 | 5.691 | < 0.001 | 0.181 |
| SLC35A4_trans | F-min | Westra et al. | -0.073 | 0.133 | -0.551 | 0.582 | 0 |
| SLC35A4_cis | F-min | Westra et al. | 0.243 | 0.048 | 5.091 | < 0.001 | 0.143 |

**Table S10.** Comparison between eQTL scores for *SLC35A4* extracted from both discovery datasets in their association with white matter tracts (MD); * = eQTL score based on significant associations in the discovery dataset for the main analysis included in manuscript.

| **eQTL score** | **White matter tract** | **Study from which score is calculated** | **Effect size** | **SD** | **t value** | **p value** | **R2 (%)** |
| --- | --- | --- | --- | --- | --- | --- | --- |
| *****PLEKHM1_cis-x | gMD | Gusev et al. | 0.034 | 0.007 | 4.600 | < 0.001 | 0.112 |
| PLEKHM1P1_cis | gMD | Westra et al. | 0.058 | 0.081 | 0.713 | 0.476 | 0 |
| *****PLEKHM1_cis-x | TR | Gusev et al. | 0.031 | 0.007 | 4.381 | < 0.001 | 0.091 |
| PLEKHM1P1_cis | TR | Westra et al. | 0.036 | 0.077 | 0.468 | 0.640 | 0 |
| *****PLEKHM1_cis-x | AF | Gusev et al. | 0.033 | 0.008 | 4.313 | < 0.001 | 0.102 |
| PLEKHM1P1_cis | AF | Westra et al. | 0.062 | 0.083 | 0.746 | 0.456 | 0 |
| *****PLEKHM1_cis-x | F-min | Gusev et al. | 0.034 | 0.008 | 4.529 | < 0.001 | 0.112 |
| PLEKHM1P1_cis | F-min | Westra et al. | -0.011 | 0.082 | -0.129 | 0.897 | 0 |
| *****PLEKHM1_cis-x | SLF | Gusev et al. | 0.035 | 0.008 | 4.661 | < 0.001 | 0 |
| PLEKHM1P1_cis | SLF | Westra et al. | 0.083 | 0.082 | 1.016 | 0.309 | 0.009 |
| *****PLEKHM1_cis-x | ATR | Gusev et al. | 0.037 | 0.007 | 5.363 | < 0.001 | 0 |
| PLEKHM1P1_cis | ATR | Westra et al. | 0.028 | 0.075 | 0.372 | 0.710 | 0.002 |

**Table S11.** Comparison between eQTL scores for *PLEKHM1* extracted from both discovery datasets in their association with white matter tracts (MD); * = eQTL score based on significant associations in the discovery dataset for the main analysis included in manuscript.

| **eQTL score** | **White matter tract** | **Study from which score is calculated** | **Effect size** | **SD** | **t value** | **p value** | **R2 (%)** |
| --- | --- | --- | --- | --- | --- | --- | --- |
| UBE3C_cis | F-min | Gusev et al. | 0.021 | 0.008 | 2.789 | 0.005 | 0.039 |
| *****UBE3C_cis | F-min | Westra et al. | 0.121 | 0.028 | 4.371 | < 0.001 | 0.104 |
| UBE3C_cis | F-min | Westra et al. | 0.155 | 0.117 | 1.322 | 0.186 | 0.004 |
| UBE3C_cis | IFOF | Gusev et al. | 0.020 | 0.007 | 2.649 | 0.008 | 0.000 |
| *****UBE3C_cis | IFOF | Westra et al. | 0.121 | 0.027 | 4.462 | < 0.001 | 0.022 |
| UBE3C_cis | IFOF | Westra et al. | 0.283 | 0.115 | 2.460 | 0.014 | 0.148 |

**Table S12.** Comparison between eQTL scores for *UBE3C* extracted from both discovery datasets in their association with white matter tracts (MD); * = eQTL score based on significant associations in the discovery dataset for the main analysis included in manuscript.

| **White Matter Tracts** | **Effect size** | **SD** | **t value** | **p value** | **p value, FDR corrected** |
| --- | --- | --- | --- | --- | --- |
| **FA** |  |  |  |  |  |
| Global FA | -0.0367 | 0.0079 | -4.6474 | 3.39 × 10^-6^ | 0.0088 |
| Thalamic radiations | -0.0403 | 0.0080 | -5.0378 | 4.76 × 10^-7^ | 0.0025 |
| Anterior thalamic radiations | -0.0429 | 0.0076 | -5.6798 | 1.37 × 10^-8^ | 0.0002 |
| Forceps minor | -0.0471 | 0.0078 | -6.0115 | 1.88 × 10^-9^ | 0.0001 |
| Superior longitudinal fasciculus | -0.0386 | 0.0077 | -5.0327 | 4.89 × 10^-7^ | 0.0040 |
| **MD** |  |  |  |  |  |
| Global MD | 0.0404 | 0.0075 | 5.3762 | 7.72 × 10^-8^ | 0.0015 |
| Association fibres | 0.0381 | 0.0077 | 4.9643 | 6.97 × 10^-7^ | 0.0045 |
| Thalamic radiations | 0.0327 | 0.0072 | 4.5625 | 5.09 × 10^-6^ | 0.0132 |
| Acoustic radiation | 0.0295 | 0.0069 | 4.2470 | 2.17 × 10^-5^ | 0.0472 |
| Anterior thalamic radiations | 0.0403 | 0.0070 | 5.7964 | 6.91 × 10^-9^ | 0.0003 |
| Cingulate gyrus | 0.0352 | 0.0073 | 4.7887 | 1.69 × 10^-6^ | 0.0085 |
| Forceps minor | 0.0480 | 0.0076 | 6.3085 | 2.89 × 10^-10^ | 2.76 × 10^-5^ |
| Inferior fronto-occipital fasciculus | 0.0410 | 0.0075 | 5.4805 | 4.31 × 10^-8^ | 0.0008 |
| Inferior longitudinal fasciculus | 0.0377 | 0.0073 | 5.1766 | 2.28 × 10^-7^ | 0.0024 |
| Superior longitudinal fasciculus | 0.0415 | 0.0076 | 5.4902 | 4.08 × 10^-8^ | 0.0008 |
| Uncinate fasciculus | 0.0314 | 0.0068 | 4.6086 | 4.08 × 10^-6^ | 0.0162 |

**Table S13**. Significant associations between DCAKD_eQTL_cis and FA and MD-measured white matter tracts.

| **White Matter Tracts** | **Effect size** | **SD** | **t value** | **p value** | **p value, FDR corrected** |
| --- | --- | --- | --- | --- | --- |
| **FA** |  |  |  |  |  |
| Global FA | -0.0403 | 0.0079 | -5.0996 | 3.44 × 10^-7^ | 0.0022 |
| Association fibres | -0.0347 | 0.0079 | -4.4036 | 1.07 × 10^-5^ | 0.0198 |
| Projection fibres | 0.0453 | 0.0079 | 5.7612 | 8.51 × 10^-9^ | 0.0002 |
| Acoustic radiation | -0.0326 | 0.0069 | -4.7044 | 2.56 × 10^-6^ | 0.0133 |
| Corticospinal tract | -0.0326 | 0.0074 | -4.3945 | 1.11 × 10^-5^ | 0.0337 |
| Forceps minor | -0.0561 | 0.0078 | -7.1754 | 7.55 × 10^-13^ | 7.32 × 10^-8^ |
| Inferior longitudinal fasciculus | -0.0335 | 0.0076 | -4.3887 | 1.14 × 10^-5^ | 0.0337 |
| Superior longitudinal fasciculus | -0.0367 | 0.0077 | -4.7887 | 1.69 × 10^-6^ | 0.0103 |
| **MD** |  |  |  |  |  |
| Global MD | 0.0308 | 0.0075 | 4.0893 | 4.35 × 10^-5^ | 0.0423 |
| Forceps minor | 0.0432 | 0.0076 | 5.6773 | 1.39 × 10^-8^ | 0.0004 |
| Inferior longitudinal fasciculus | 0.0362 | 0.0073 | 4.9676 | 6.85 × 10^-7^ | 0.0044 |

**Table S14.** Significant associations between SLC35A4_eQTL_cis and FA and MD-measured white matter tracts.

| **White Matter Tracts** | **Effect size** | **SD** | **t value** | **p value** | **p value, FDR corrected** |
| --- | --- | --- | --- | --- | --- |
| **FA** |  |  |  |  |  |
| Global FA | -0.0420 | 0.0079 | -5.3199 | 1.05 × 10^-7^ | 0.0011 |
| Association fibres | -0.0358 | 0.0079 | -4.5425 | 5.60 × 10^-6^ | 0.0121 |
| Thalamic radiations | -0.0388 | 0.0080 | -4.8429 | 1.29 × 10^-6^ | 0.0048 |
| Projection fibres | 0.0416 | 0.0079 | 5.2850 | 1.27 × 10^-7^ | 0.0011 |
| Corticospinal tract | -0.0320 | 0.0074 | -4.3116 | 1.63 × 10^-5^ | 0.0416 |
| Forceps minor | -0.0456 | 0.0078 | -5.8270 | 5.76 × 10^-9^ | 0.0001 |
| Inferior longitudinal fasciculus | -0.0419 | 0.0076 | -5.4773 | 4.39 × 10^-8^ | 0.0006 |
| Posterior thalamic radiation | -0.0352 | 0.0075 | -4.7014 | 2.60 × 10^-6^ | 0.0133 |
| Superior longitudinal fasciculus | -0.0392 | 0.0077 | -5.1143 | 3.18 × 10^-7^ | 0.0028 |
| **MD** |  |  |  |  |  |
| Global MD | 0.0326 | 0.0075 | 4.3299 | 1.50 × 10^-5^ | 0.0277 |
| Acoustic radiation | 0.0339 | 0.0069 | 4.8778 | 1.08 × 10^-6^ | 0.0060 |
| Cingulate gyrus | 0.0328 | 0.0073 | 4.4648 | 8.07 × 10^-6^ | 0.0248 |
| Forceps minor | 0.0348 | 0.0076 | 4.5604 | 5.14 × 10^-6^ | 0.0188 |

**Table S15.** Significant associations between SEC14L4_eQTL_cis and FA and MD-measured white matter tracts.

| **White Matter Tracts** | **Effect size** | **SD** | **t value** | **p value** | **p value, FDR corrected** |
| --- | --- | --- | --- | --- | --- |
| **FA** |  |  |  |  |  |
| Projection fibres | 0.0339 | 0.0079 | 4.3032 | 1.69 × 10^-5^ | 0.0273 |
| Forceps minor | -0.0462 | 0.0078 | -5.8981 | 3.75 × 10^-9^ | 0.0001 |
| **MD** |  |  |  |  |  |
| Forceps minor | 0.0353 | 0.0076 | 4.6349 | 3.60 × 10^-6^ | 0.0155 |

**Table S16.** Significant associations between SRA1_eQTL_cis and FA and MD-measured white matter tracts.

| **White Matter Tracts** | **Effect size** | **SD** | **t value** | **p value** | **p value, FDR corrected** |
| --- | --- | --- | --- | --- | --- |
| **FA** |  |  |  |  |  |
| Anterior thalamic radiations | 0.0324 | 0.0076 | 4.2863 | 1.82 × 10^-5^ | 0.0429 |
| Forceps minor | 0.0352 | 0.0078 | 4.4956 | 6.99 × 10^-6^ | 0.0271 |
| **MD** |  |  |  |  |  |
| Global MD | -0.0328 | 0.0075 | -4.3626 | 1.29 × 10^-5^ | 0.0257 |
| Anterior thalamic radiations | -0.0339 | 0.0070 | -4.8703 | 1.12 × 10^-6^ | 0.0060 |
| Forceps minor | -0.0392 | 0.0076 | -5.1537 | 2.58 × 10^-7^ | 0.0025 |
| Inferior fronto-occipital fasciculus | -0.0335 | 0.0075 | -4.4845 | 7.36 × 10^-6^ | 0.0234 |
| Inferior longitudinal fasciculus | -0.0311 | 0.0073 | -4.2695 | 1.97 × 10^-5^ | 0.0447 |
| Superior longitudinal fasciculus | -0.0343 | 0.0076 | -4.5355 | 5.79 × 10^-6^ | 0.0204 |

**Table S17.** Significant associations between NMT1_eQTL_cis and FA and MD-measured white matter tracts.

| **White Matter Tracts** | **Effect size** | **SD** | **t value** | **p value** | **p value, FDR corrected** |
| --- | --- | --- | --- | --- | --- |
| **FA** |  |  |  |  |  |
| Forceps major | 0.0436 | 0.0081 | 5.3818 | 7.49 × 10^-8^ | 0.0009 |
| Forceps minor | 0.0338 | 0.0078 | 4.3185 | 1.58 × 10^-5^ | 0.0416 |
| **MD** |  |  |  |  |  |
| Global MD | -0.0366 | 0.0075 | -4.8650 | 1.15 × 10^-6^ | 0.0050 |
| Association fibres | -0.0368 | 0.0077 | -4.7868 | 1.71 × 10^-6^ | 0.0063 |
| Inferior longitudinal fasciculus | -0.0309 | 0.0073 | -4.2303 | 2.34 × 10^-5^ | 0.0497 |
| Superior longitudinal fasciculus | -0.0356 | 0.0076 | -4.7055 | 2.55 × 10^-6^ | 0.0116 |

**Table S18.** Significant associations between CPNE1_eQTL_cis and FA and MD-measured white matter tracts.

| **White Matter Tracts** | **Effect size** | **SD** | **t value** | **p value** | **p value, FDR corrected** |
| --- | --- | --- | --- | --- | --- |
| **FA** |  |  |  |  |  |
| Forceps minor | -0.0347 | 0.0078 | -4.4321 | 9.40 × 10^-6^ | 0.0337 |
| **MD** |  |  |  |  |  |
| Global MD | 0.0330 | 0.0075 | 4.3859 | 1.16 × 10^-5^ | 0.0250 |
| Association fibres | 0.0318 | 0.0077 | 4.1395 | 3.50 × 10^-5^ | 0.0423 |
| Thalamic radiations | 0.0296 | 0.0072 | 4.1282 | 3.67 × 10^-5^ | 0.0423 |
| Anterior thalamic radiations | 0.0356 | 0.0070 | 5.1101 | 3.26 × 10^-7^ | 0.0028 |
| Forceps minor | 0.0334 | 0.0076 | 4.3876 | 1.15 × 10^-5^ | 0.0297 |
| Superior longitudinal fasciculus | 0.0342 | 0.0076 | 4.5223 | 6.16 × 10^-6^ | 0.0210 |

**Table S19.** Significant associations between PLEKHM1_eQTL_cis and FA and MD-measured white matter tracts.

| **White Matter Tracts** | **Effect size** | **SD** | **t value** | **p value** | **p value, FDR corrected** |
| --- | --- | --- | --- | --- | --- |
| **FA** |  |  |  |  |  |
| Forceps minor | -0.0382 | 0.0078 | -4.8721 | 1.11 × 10^-6^ | 0.0077 |
| **MD** |  |  |  |  |  |
| Forceps minor | 0.0331 | 0.0076 | 4.3465 | 1.39 × 10^-5^ | 0.0349 |
| Inferior fronto-occipital fasciculus | 0.0332 | 0.0075 | 4.4413 | 9.01 × 10^-6^ | 0.0268 |

**Table S20.** Significant associations between UBE3C_eQTL_cis and FA and MD-measured white matter tracts.

| **Score, white matter tract** | **Effect size** | **SD** | **t value** | **p value** | **p value, FDR corrected** |
| --- | --- | --- | --- | --- | --- |
| ATG10_eQTL_cis, global FA | 0.0341 | 0.0079 | 4.3106 | 1.64 × 10^-5^ | 0.0273 |
| SF3A1_eQTL_cis, global FA | -0.0327 | 0.0079 | -4.1305 | 3.64 × 10^-5^ | 0.0495 |
| SMARCAL1_eQTL_cis, global FA | 0.0374 | 0.0079 | 4.7354 | 2.21 × 10^-6^ | 0.0071 |
| SF3A1_eQTL_cis, association fibres | -0.0334 | 0.0079 | -4.2386 | 2.26 × 10^-5^ | 0.0344 |
| SMARCAL1_eQTL_cis, association fibres | 0.0326 | 0.0079 | 4.1375 | 3.53 × 10^-5^ | 0.0495 |
| ATG10_eQTL_cis, thalamic radiations | 0.0373 | 0.0080 | 4.6587 | 3.21 × 10^-6^ | 0.0088 |
| PPP4R3A_eQTL_cis, thalamic radiations | 0.0357 | 0.0080 | 4.4572 | 8.36 × 10^-6^ | 0.0166 |
| SMARCAL1_eQTL_cis, thalamic radiations | 0.0394 | 0.0080 | 4.9292 | 8.35 × 10^-7^ | 0.0036 |
| CD14_eQTL_cis, projection fibres | -0.0360 | 0.0079 | -4.5691 | 4.94 × 10^-6^ | 0.0116 |
| COG7_eQTL_cis, anterior thalamic radiation | -0.0333 | 0.0076 | -4.4005 | 1.09 × 10^-5^ | 0.0337 |
| SMARCAL1_eQTL_cis, anterior thalamic radiation | 0.0394 | 0.0076 | 5.2164 | 1.85 × 10^-7^ | 0.0018 |
| LINC01605_eQTL_trans, cingulate gyrus | -0.0337 | 0.0071 | -4.7560 | 1.99 × 10^-6^ | 0.0114 |
| ANXA1_eQTL_cis, corticospinal tract | -0.0320 | 0.0074 | -4.3218 | 1.56 × 10^-5^ | 0.0416 |
| ZSCAN26_eQTL_cis, forceps major | -0.0397 | 0.0081 | -4.9048 | 9.45 × 10^-7^ | 0.0070 |
| ATG10_eQTL_cis, forceps minor | 0.0360 | 0.0078 | 4.5986 | 4.29 × 10^-6^ | 0.0189 |
| CD14_eQTL_cis, forceps minor | 0.0456 | 0.0078 | 5.8210 | 6 × 10^-9^ | 0.0001 |
| SHTN1 / KIAA1598_eQTL_cis, forceps minor | 0.0376 | 0.0078 | 4.8050 | 1.56 × 10^-6^ | 0.0101 |
| ZNF282_eQTL_cis, forceps minor | -0.0346 | 0.0078 | -4.4224 | 9.83 × 10^-6^ | 0.0337 |
| ENO4_eQTL_cis, forceps minor | 0.0354 | 0.0078 | 4.5197 | 6.24 × 10^-6^ | 0.0252 |
| COG7_eQTL_cis, forceps minor | -0.0338 | 0.0078 | -4.3127 | 1.62 × 10^-5^ | 0.0416 |
| SMARCAL1_eQTL_cis, forceps minor | 0.0361 | 0.0078 | 4.6056 | 4.15 × 10^-6^ | 0.0189 |
| ASRGL1_eQTL_cis, inferior fronto-occipital fasciculus | 0.0329 | 0.0077 | 4.2950 | 1.76 × 10^-5^ | 0.0426 |
| ATG10_eQTL_cis, inferior fronto-occipital fasciculus | 0.0355 | 0.0077 | 4.6291 | 3.7 × 10^-6^ | 0.0179 |
| TMEM184B_eQTL_cis, inferior fronto-occipital fasciculus | 0.0337 | 0.0077 | 4.3935 | 1.12 × 10^-5^ | 0.0337 |
| SMARCAL1_eQTL_cis, inferior longitudinal fasciculus | 0.0349 | 0.0076 | 4.5704 | 4.91 × 10^-6^ | 0.0207 |
| ATG10_eQTL_cis, posterior thalamic radiation | 0.0325 | 0.0075 | 4.3416 | 1.42 × 10^-5^ | 0.0406 |
| ZBTB7B_eQTL_cis, superior longitudinal fasciculus | -0.0329 | 0.0077 | -4.2946 | 1.76 × 10^-5^ | 0.0426 |
| GPT_eQTL_cis, superior longitudinal fasciculus | 0.0339 | 0.0077 | 4.4153 | 1.02 × 10^-5^ | 0.0337 |
| SMARCAL1_eQTL_cis, superior longitudinal fasciculus | 0.0401 | 0.0077 | 5.2356 | 1.67 × 10^-7^ | 0.0018 |
| GPT_eQTL_cis, superior thalamic radiation | 0.0337 | 0.0079 | 4.2827 | 1.86 × 10^-5^ | 0.0429 |
| AP2S1_eQTL_cis, superior thalamic radiation | 0.0348 | 0.0079 | 4.4164 | 1.01 × 10^-5^ | 0.0337 |

**Table S21.** eQTL scores associated only with white matter tracts as measured by FA.

| **Score, white matter tract** | **Effect size** | **SD** | **t value** | **p value** | **p value, FDR corrected** |
| --- | --- | --- | --- | --- | --- |
| APOA1BP / NAXE_eQTL_cis, global MD | 0.0311 | 0.0075 | 4.1331 | 3.6 × 10^-5^ | 0.0423 |
| BTN3A2_eQTL_cis, global MD | 0.0308 | 0.0075 | 4.0853 | 4.42 × 10^-5^ | 0.0423 |
| UMPS_eQTL_cis, global MD | -0.0319 | 0.0075 | -4.2381 | 2.27 × 10^-5^ | 0.0366 |
| CSF3R_eQTL_cis, global MD | 0.0345 | 0.0075 | 4.5704 | 4.91 × 10^-6^ | 0.0132 |
| TMEM154_eQTL_cis, global MD | -0.0400 | 0.0076 | -5.2970 | 1.19 × 10^-7^ | 0.0015 |
| APOA1BP / NAXE_eQTL_cis, association fibres | 0.0326 | 0.0077 | 4.2419 | 2.23 × 10^-5^ | 0.0366 |
| BTN3A2_eQTL_cis, association fibres | 0.0314 | 0.0077 | 4.0888 | 4.36 × 10^-5^ | 0.0423 |
| SAMM50_eQTL_cis, association fibres | -0.0311 | 0.0077 | -4.0501 | 5.15 × 10^-5^ | 0.0475 |
| UMPS_eQTL_cis, association fibres | -0.0355 | 0.0077 | -4.6219 | 3.84 × 10^-6^ | 0.0124 |
| CSF3R_eQTL_cis, association fibres | 0.0377 | 0.0077 | 4.9069 | 9.36 × 10^-7^ | 0.0048 |
| TMEM154_eQTL_cis, association fibres | -0.0402 | 0.0077 | -5.2158 | 1.86 × 10^-7^ | 0.0016 |
| HLA-C_eQTL_cis, association fibres | -0.0342 | 0.0077 | -4.4402 | 9.05 × 10^-6^ | 0.0213 |
| MED15_eQTL_cis, thalamic radiations | 0.0297 | 0.0072 | 4.1354 | 3.56 × 10^-5^ | 0.0423 |
| KANSL1_eQTL_cis, thalamic radiations | -0.0302 | 0.0072 | -4.2041 | 2.64 × 10^-5^ | 0.0401 |
| IL18RAP_eQTL_cis, projection fibres | 0.0324 | 0.0078 | 4.1621 | 3.17 × 10^-5^ | 0.0423 |
| C6orf106_eQTL_cis, projection fibres | 0.0318 | 0.0078 | 4.0863 | 4.41 × 10^-5^ | 0.0423 |
| RABEPK_eQTL_cis, acoustic radiation | 0.0307 | 0.0069 | 4.4197 | 9.95 × 10^-6^ | 0.0287 |
| CFDP1_eQTL_cis, anterior thalamic radiation | 0.0298 | 0.0070 | 4.2749 | 1.92 × 10^-5^ | 0.0447 |
| PTPN13_eQTL_cis, anterior thalamic radiation | -0.0347 | 0.0070 | -4.9756 | 6.58 × 10^-7^ | 0.0044 |
| KANSL1_eQTL_cis, anterior thalamic radiation | -0.0369 | 0.0070 | -5.3005 | 1.17 × 10^-7^ | 0.0016 |
| TMEM154_eQTL_cis, anterior thalamic radiation | -0.0303 | 0.0070 | -4.3269 | 1.52 × 10^-5^ | 0.0372 |
| UMPS_eQTL_cis, cingulate gyrus | -0.0323 | 0.0073 | -4.3999 | 1.09 × 10^-5^ | 0.0297 |
| PLEC_eQTL_cis, forceps minor | 0.0335 | 0.0076 | 4.3887 | 1.15 × 10^-5^ | 0.0297 |
| TMEM154_eQTL_cis, forceps minor | -0.0384 | 0.0076 | -5.0145 | 5.38 × 10^-7^ | 0.0043 |
| TMEM154_eQTL_cis, inferior fronto-occipital fasciculus | -0.0330 | 0.0075 | -4.3971 | 1.1 × 10^-5^ | 0.0297 |
| TMEM154_eQTL_cis, inferior longitudinal fasciculus | -0.0337 | 0.0073 | -4.5974 | 4.32 × 10^-6^ | 0.0164 |
| SAMM50_eQTL_cis, parahippocampal part of cingulum | -0.0303 | 0.0071 | -4.2755 | 1.92 × 10^-5^ | 0.0447 |
| BTN3A2_eQTL_cis, superior longitudinal fasciculus | 0.0350 | 0.0076 | 4.6271 | 3.74 × 10^-6^ | 0.0155 |
| UMPS_eQTL_cis, superior longitudinal fasciculus | -0.0413 | 0.0076 | -5.4562 | 4.94 × 10^-8^ | 0.0008 |
| TMEM154_eQTL_cis, superior longitudinal fasciculus | -0.0377 | 0.0076 | -4.9633 | 7.01 × 10^-7^ | 0.0044 |
| PTPN13_eQTL_trans, superior thalamic radiation | -0.0336 | 0.0068 | -4.9424 | 7.8 × 10^-7^ | 0.0046 |
| TMEM154_eQTL_cis, superior thalamic radiation | -0.0323 | 0.0068 | -4.7333 | 2.23 × 10^-6^ | 0.0106 |
| TMEM154_eQTL_cis, uncinate fasciculus | -0.0308 | 0.0068 | -4.5058 | 6.66 × 10^-6^ | 0.0219 |

**Table S22.** eQTL scores associated only with white matter tracts as measured by MD.

|  | | | | |
| --- | --- | --- | --- | --- |
| **Score name & eQTL type** | **N SNPs in score** | **Regulated gene** | **Study from which score is calculated** | **Gene function** |
| ATG10_eQTL_cis | 7 | ATG10 | Gusev et al. | E2-like enzyme involved in 2 ubiquitin-like modifications essential for autophagosome formation; expressed in brain (1) |
| SF3A1_eQTL_cis | 23 | SF3A1 | Westra et al. | Expressed in brain; gene encodes a subunit of the splicing factor 3a protein complex (2) |
| SMARCAL1_eQTL_cis | 1 | SMARCAL1 | Westra et al. | Protein encoded by this gene is a member of SWI/SNP family of proteins; members have helicase and ATPase activities and are thought to regulate transcription of certain genes by altering chromatin structure around those genes; expressed in brain; associated with Schimke immunoosseous dysplasia (3) |
| PPP4R3A_eQTL_cis | 1 | PPP4R3A | Westra et al, | Expressed in brain; may be involved in Alzheimer’s disease risk (4) |
| CD14_eQTL_cis | 18 | CD14 | Gusev et al. | Protein encoded by this gene is a surface antigen that is preferentially expressed on monocytes/macrophages; it cooperates with other proteins to mediate the innate immune response to bacterial lipopolysaccharide; expressed in brain (5) |
| COG7_eQTL_cis | 1 | COG7 | Westra et al. | [Protein encoded by this gene resides in the golgi and is part of 8 subunits of the conserved oligomeric Golgi (COG) complex; expressed in brain; mutations in gene associated with microcephaly, adducted thumbs, growth retardation, VSD and episodes of hyperthermia (6; 7)](Protein%20encoded%20by%20this%20gene%20resides%20in%20the%20golgi%20and%20is%20part%20of%208%20subunits%20of%20the%20conserved%20oligomeric%20Golgi%20(COG)%20complex;%20expressed%20in%20brain;%20) |
| LINC01605_eQTL_trans | 1 | LINC01605 | Westra et al. | [RNA gene; affiliated with non-coding RNA class; expression of gene associated with bladder cancer](http://www.bioscirep.org/content/early/2018/07/27/BSR20180562) (8) |
| ANXA4_eQTL_cis | 5 | ANXA4 | Gusev et al. | Little expression in brain; gene belongs to annexin family of calcium dependent phospholipid binding proteins (9) |
| ZSCAN26_eQTL_cis | 32 | ZSCAN26 | Westra et al. | Expressed in brain; protein coding gene |
| SHTN1 / KIAA1598_eQTL_cis | 5 | SHTN1 / KIAA1598 | Gusev et al. | [Expressed in brain; involved in generation of internal asymmetric signals required for neuronal polarization and neurite outgrowth; mediated netrin-1-induced F-actin substrate coupling or clutch engagement within axon growth cone through activation of several genes & pathways](https://www.ncbi.nlm.nih.gov/pubmed/18519736/) (10) |
| ZNF282_eQTL_cis | 7 | ZNF282 | Gusev et al. | Expressed in brain; diseases associated with gene: T-cell leukemia (11) |
| ENO4_eQTL_cis | 7 | ENO4 | Westra et al. | Expressed in brain |
| ASRGL1_eQTL_cis | 5 | ASRGL1 | Gusev et al. | Expressed in brain; may be involved in production of L-aspartate, which can act as an excitatory neurotransmitter in some brain regions; may be implicated in endometrioid endometrial carcinoma (12) |
| TMEM184B_eQTL_cis | 12 | TMEM184B | Gusev et al. | Expressed in brain; may be implicated in axon degeneration (13) |
| ZBTB7B_eQTL_cis | 8 | ZBTB7B | Gusev et al. | Expressed in brain; gene encodes a zinc finger-containing transcription factor that acts as a key regulator of lineage commitment of immature T-cell precursors (14) |
| GPT_eQTL_cis | 5 | GPT | Westra et al. | Little expression in brain |
| AP2S1_eQTL_cis | 5 | AP2S1 | Westra et al. | One of 2 major clathrin-associated adaptor complexes, AP-2 is a heterotetramer which is associated with the plasma membrane; complex is composed of 2 large chains, 1 medium chain and 1 small chain, and the gene encodes the small chain; expressed in brain (15) |

**Table S23**. Information regarding eQTL scores with significant associations FA-measured tracts. 1 score (LINC01605_eQTL_trans) is trans, while all others are cis.

| **Score name & eQTL type** | **N SNPs in score** | **Regulated gene** | **Study from which score is calculated** | **Gene function** |
| --- | --- | --- | --- | --- |
| APOA1BP / NAXE_eQTL_cis | 10 | APOA1BP / NAXE | Gusev et al. | [Expressed in brain; diseases associated with gene: encephalopathy; brain edema](https://www.ncbi.nlm.nih.gov/pubmed/27616477/) (16) |
| BTN3A2_eQTL_cis | 42 | BTN3A2 | Gusev et al. | May be involved in adaptive immune system response; may be involved in risk for gastric cancer (17) |
| UMPS_eQTL_cis | 5 | UMPS | Westra et al. | Encoded protein is a bifunctional enzyme that catalyzes the final 2 steps of the de novo pyrimidine biosynthetic pathway (18) |
| CSF3R_eQTL_cis | 5 | CSF3R | Westra et al. | Mutations in this gene are a cause of Kostmann syndrome / congenital neutropenia; not expressed in brain (19) |
| TMEM154_eQTL_cis | 20 | TMEM154 | Westra et al. | Very little expression in brain |
| SAMM50_eQTL_cis | 15 | SAMM50 | Gusev et al. | Gene encodes a component of the Sorting and Assembly Machinery of the mitochondrial outer membrane (20) |
| HLA-C_eQTL_cis | 38 | HLA-C | Westra et al. | Expressed in nearly all cells |
| MED15_eQTL_cis | 6 | MED15 | Westra et al. | Expressed in brain |
| KANSL1_eQTL_cis | 3 | KANSL1 | Westra et al. | [Gene encodes a nuclear protein that is a subunit of 2 protein complexes involved with histone acetylation](https://bmcmedgenet.biomedcentral.com/articles/10.1186/s12881-015-0211-0) (21) |
| IL18RAP_eQTL_cis | 12 | IL18RAP | Gusev et al. | Mutations in this gene have been associated with Crohn's disease; expressed in brain (22) |
| C6orf106_eQTL_cis | 5 | C6orf106 | Westra et al. | [Expressed in cortex](https://www.proteinatlas.org/ENSG00000196821-C6orf106/tissue) |
| RABEPK_eQTL_cis | 9 | RABEPK | Gusev et al. | Expressed in brain |
| CFDP1_eQTL_cis | 4 | CFDP1 | Gusev et al. | [Expressed in brain; may be implicated in coronary artery disease risk](https://www.malacards.org/card/acute_stress_disorder) (23) |
| PTPN13_eQTL_trans | 1 | PTPN13 | Westra et al. | [Protein encoded by this gene is a member of the PTP family, which are signalling molecules that regulate cellular processes (e.g. cell growth, differentiation, mitotic cell cycle, oncogenic transformation); disease associated with this gene: tropical spastic paraparesis, a disease of the nervous system affecting people living near the equator; expressed in the brain](https://www.malacards.org/card/tropical_spastic_paraparesis) (24) |
| EVL_eQTL_cis | 1 | EVL | Westra et al. | Expressed in brain; actin-associated proteins involved in proccesses such as axon guidance and lamellipodial and filopodial dynamics in migrating cells; enhances actin nucleation and polymerization (25) |
| PLEC_eQTL_cis | 3 | PLEC | Westra et al. | Prominent member of a protein family of proteins which interlink different elements of the cytoskeleton; expressed in a wide range of cell types and tissues (including brain) (26) |

**Table S24**. Information regarding eQTL scores with significant associations MD-measured tracts. 1 score (PTPN13_eQTL_trans) is trans, while all others are cis.

| **Allen Brain Atlas Donor** | **Demographic characteristics** |
| --- | --- |
| H0351.2001 | 24 years, Male, African American |
| H0351.2002 | 39 years, Male, African American |
| H0351.1009 | 57 years, Male, Caucasian |
| H0351.1012 | 31 years, Male, Caucasian |
| H0351.1015 | 49 years, Female, Hispanic |
| H0351.1016 | 55 years, Male, Caucasian |
| **Table S25**. Descriptive statistics of the 6 donors included in the Allen Brain Atlas (29). | |

| **Chromosome** | **SNP** | **Gene** | **Effect size** | **P-value** |
| --- | --- | --- | --- | --- |
| 17 | rs4793119 | DCAKD | 0.188 | 3.98 × 10^-8^ |
| 17 | rs17682536 | DCAKD | -0.114 | 1.20 × 10^-13^ |
| 17 | rs962888 | DCAKD | 0.284 | 4.14 × 10^-19^ |
| 17 | rs9898793 | DCAKD | 0.343 | 3.53 × 10^-240^ |
| 17 | rs2040558 | DCAKD | -0.171 | 1.73 × 10^-52^ |
| 17 | rs2239921 | DCAKD | 0.367 | 1.03 × 10^-28^ |
| 17 | rs3744760 | DCAKD | 0.365 | 2.47 × 10^-184^ |
| 17 | rs4986172 | DCAKD | 0.093 | 1.07 × 10^-24^ |
| 5 | rs269783 | SLC35A4 | -0.114 | 6.55 × 10^-11^ |
| 5 | rs13175916 | SLC35A4 | -0.025 | 1.33 × 10^-9^ |
| 5 | rs2237077 | SLC35A4 | 0.322 | < 0.001 |
| 5 | rs1862176 | SLC35A4 | 0.223 | < 0.001 |
| 5 | rs6860077 | SLC35A4 | 0.210 | < 0.001 |
| 5 | rs17286676 | SLC35A4 | -0.041 | 9.04 × 10^-97^ |
| 5 | rs250430 | SLC35A4 | 0.087 | 4.09 × 10^-86^ |
| 5 | rs250429 | SLC35A4 | 0.208 | < 0.001 |
| 5 | rs12517200 | SLC35A4 | 0.061 | 7.19 × 10^-298^ |
| 5 | rs1583005 | SLC35A4 | 0.138 | 5.45 × 10^-6^ |
| 5 | rs2286394 | SLC35A4 | -0.055 | 4.40 × 10^-39^ |
| 5 | rs3733709 | SLC35A4 | -0.110 | 5.77 × 10^-24^ |
| 22 | rs2267161 | SEC14L4 | -0.093 | 6.96 × 10^-6^ |
| 5 | rs2237077 | SRA1 | -0.025 | 4.55 × 10^-19^ |
| 5 | rs1862176 | SRA1 | -0.040 | 1.72 × 10^-39^ |
| 5 | rs6860077 | SRA1 | -0.039 | 3.91 × 10^-41^ |
| 5 | rs1835959 | SRA1 | -0.087 | 1.14 × 10^-20^ |
| 5 | rs250430 | SRA1 | -0.087 | 1.12 × 10^-20^ |
| 5 | rs250429 | SRA1 | -0.039 | 2.05 × 10^-42^ |
| 5 | rs2569163 | SRA1 | 0.048 | 3.13 × 10^-24^ |
| 5 | rs778582 | SRA1 | -0.016 | 2.08 × 10^-34^ |
| 5 | rs12517200 | SRA1 | -0.013 | 6.77 × 10^-34^ |
| 5 | rs1583005 | SRA1 | -0.005 | 1.10 × 10^-11^ |
| 5 | rs2530241 | SRA1 | 0.001 | 3.92 × 10^-14^ |
| 5 | rs801186 | SRA1 | 0.010 | 3.28 × 10^-18^ |
| 5 | rs801171 | SRA1 | 0.001 | 5.85 × 10^-15^ |
| 5 | rs2531360 | SRA1 | 0.000 | 6.17 × 10^-14^ |
| 5 | rs2240696 | SRA1 | -0.007 | 6.31 × 10^-12^ |
| 17 | rs9898793 | NMT1 | 0.015 | 3.69 × 10^-8^ |
| 17 | rs4793172 | NMT1 | 0.029 | 1.41 × 10^-10^ |
| 17 | rs2239916 | NMT1 | 0.035 | 1.17 × 10^-14^ |
| 17 | rs1053739 | NMT1 | 0.032 | 2.77 × 10^-13^ |
| 17 | rs3744760 | NMT1 | 0.032 | 2.85 × 10^-10^ |
| 17 | rs12946454 | NMT1 | 0.028 | 8.33 × 10^-9^ |
| 17 | rs4986172 | NMT1 | 0.030 | 5.50 × 10^-9^ |
| 6 | rs4324798 | CPNE1 | 0.181 | 1.79 × 10^-6^ |
| 17 | rs9898793 | PLEKHM1 | 0.394 | 6.38 × 10^-78^ |
| 17 | rs2239921 | PLEKHM1 | 0.304 | 6.23 × 10^-13^ |
| 17 | rs3744760 | PLEKHM1 | 0.247 | 2.54 × 10^-79^ |
| 17 | rs4986172 | PLEKHM1 | -0.138 | 3.73 × 10^-6^ |
| 17 | rs1552458 | PLEKHM1 | 0.317 | 2.91 × 10^-47^ |
| 7 | rs17646960 | UBE3C | 0.342 | 1.49 × 10^-20^ |
| 7 | rs1182398 | UBE3C | -0.285 | 1.07 × 10^-33^ |
| 7 | rs1182393 | UBE3C | -0.298 | 9.37 × 10^-43^ |
| 7 | rs2527866 | UBE3C | -0.297 | 2.95 × 10^-6^ |

**Table S26**. Associations between SNPs found in the 8 eQTL scores and gene expression (N_total_ = 53); effect size and p-values are taken from the two GWAS studies (Gusev et al., 2016; Westra et al., 2013) available in GENOSCORES.

**
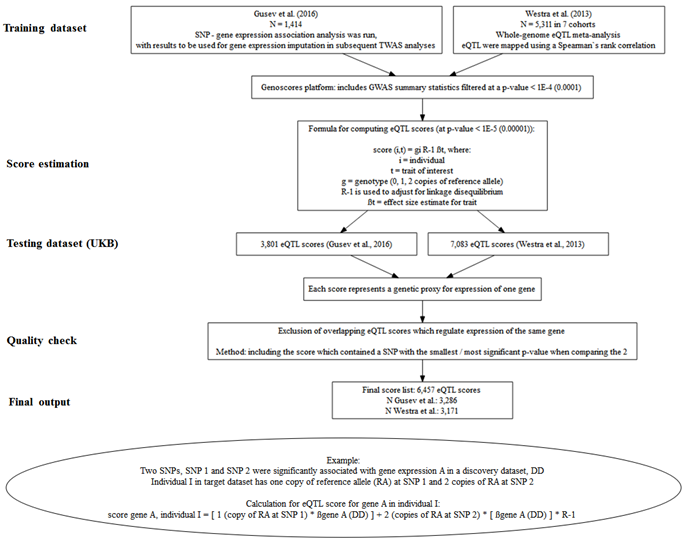
**

**Figure S1.** eQTL score computation process.


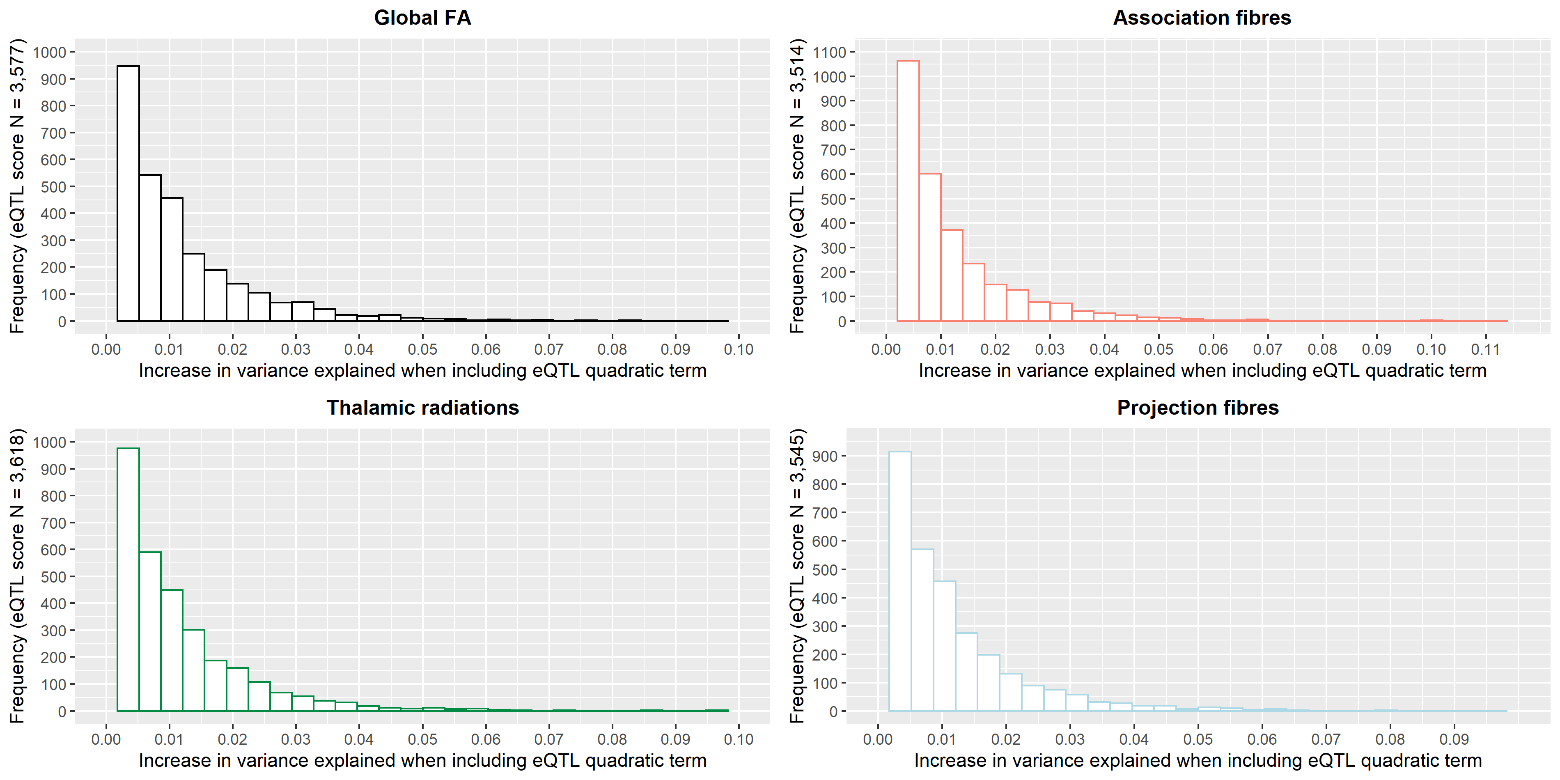
**Figure S2.** Frequency of eQTL scores that increased variance explained in FA global and tract category measures; the x-axis indicates increase in variance explained (%) when including the eQTL quadratic terms; the y-axis indicates the number of eQTL quadratic terms that increase the variance explained.


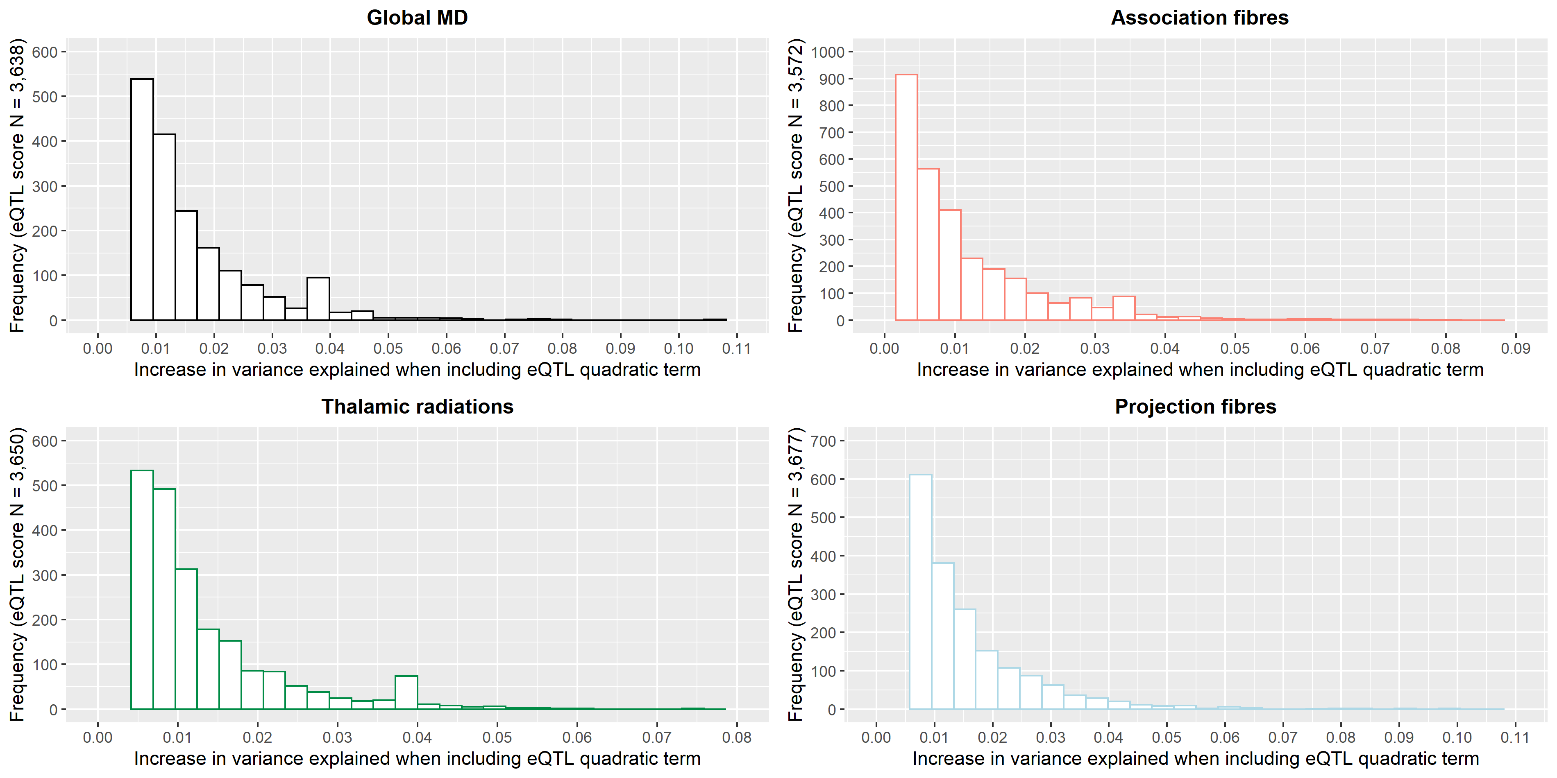


**Figure S3.** Frequency of eQTL scores that increased variance explained in MD global and tract category measures; the x-axis indicates increase in variance explained (%) when including the eQTL quadratic terms; the y-axis indicates the number of eQTL quadratic terms that increase the variance explained.

**
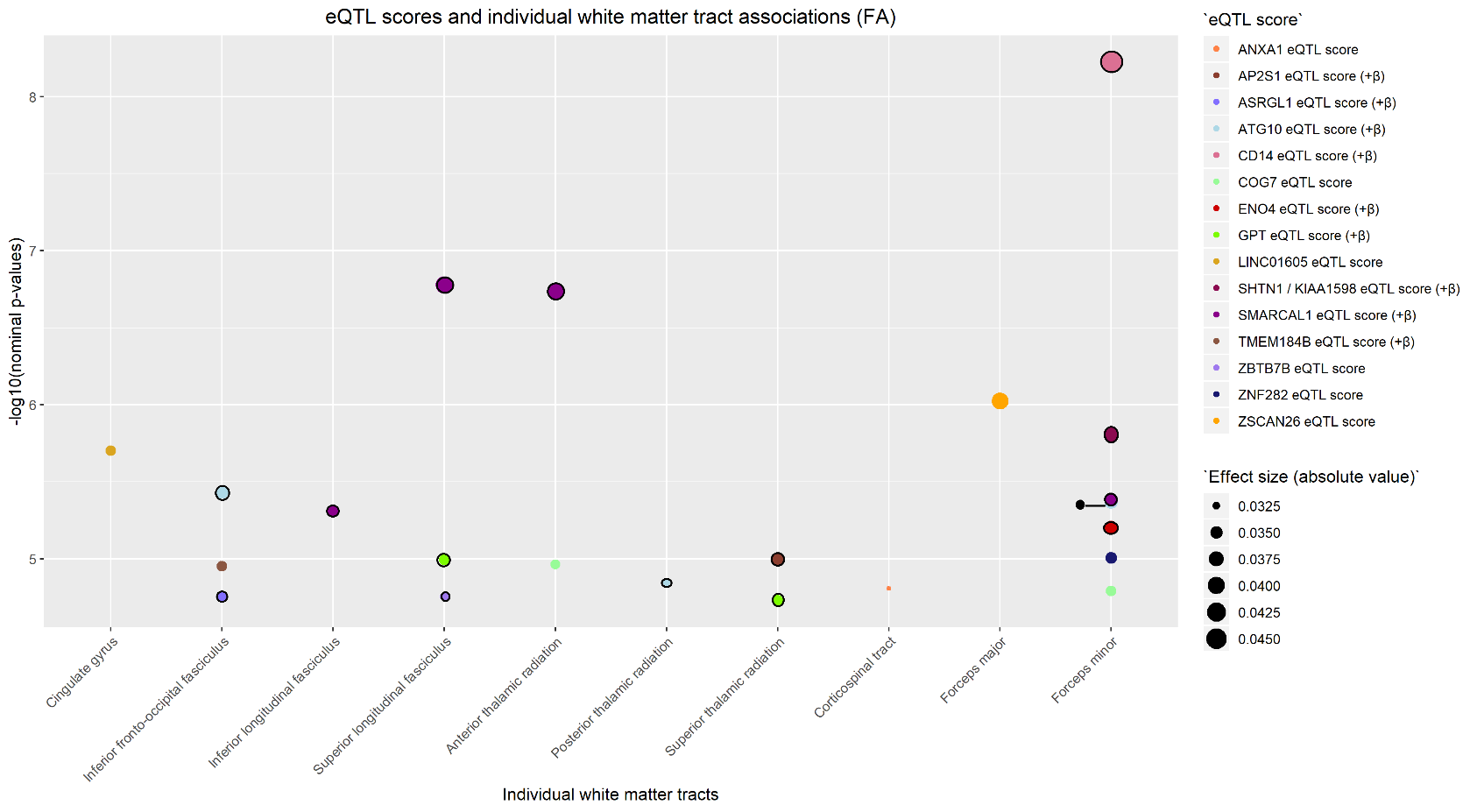

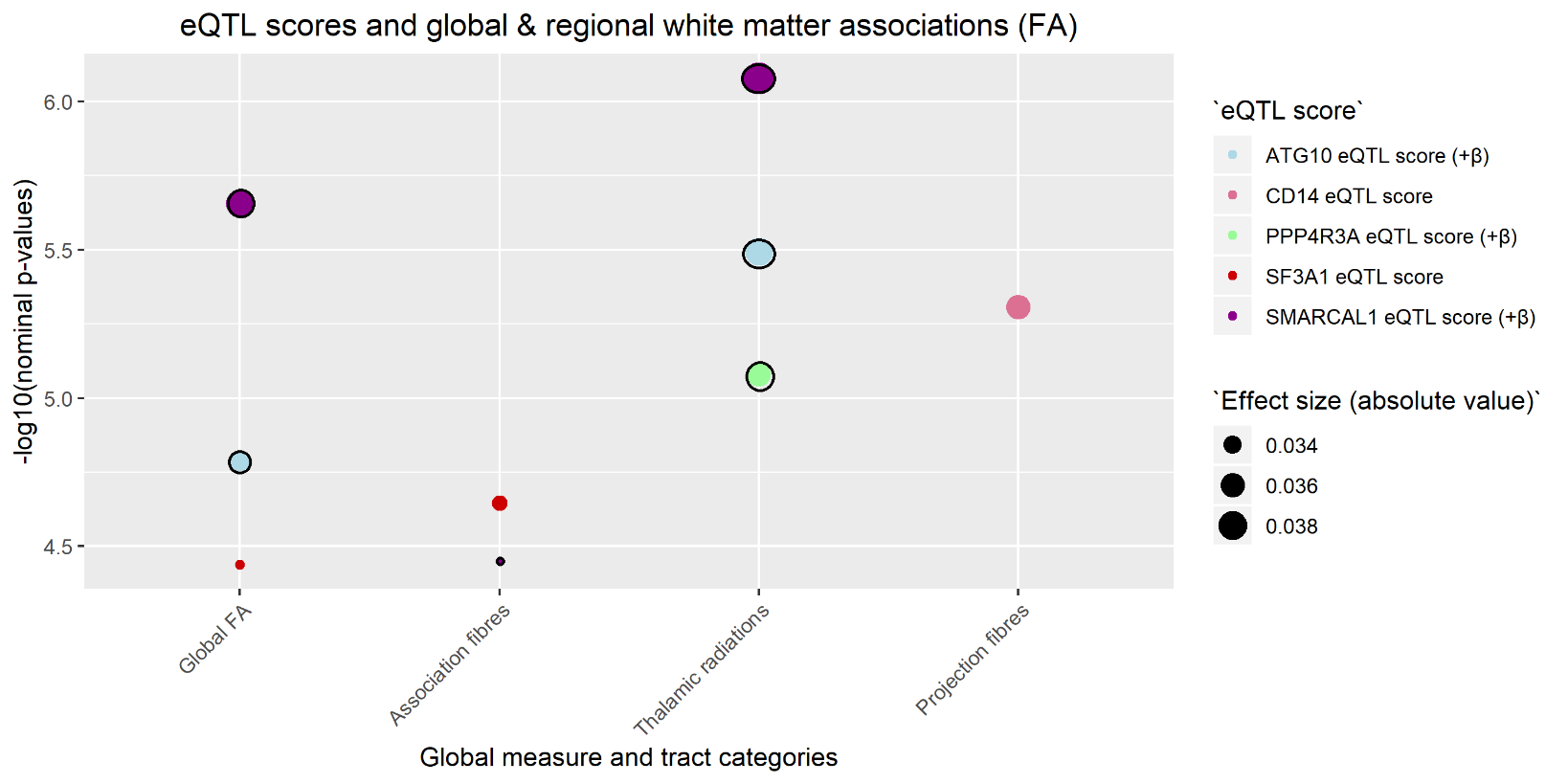
**

**Figure S4.** eQTL scores associated only with white matter tracts as measured by FA (fractional anisotropy). Indicates nominal p-values between each of the scores (shown in legend entitled “eQTL score”) and global and tract category measures (noted on the x-axis). All values in the figure met FDR correction. Some of the scores with an additional line around the points had an effect size in the opposite direction to all other scores (also indicated by +β for FA in figure legend). The colours of the plot points indicate the score to which they belong. Magnitude of effect is shown in the legend entitled “Effect size (absolute values)”.

**
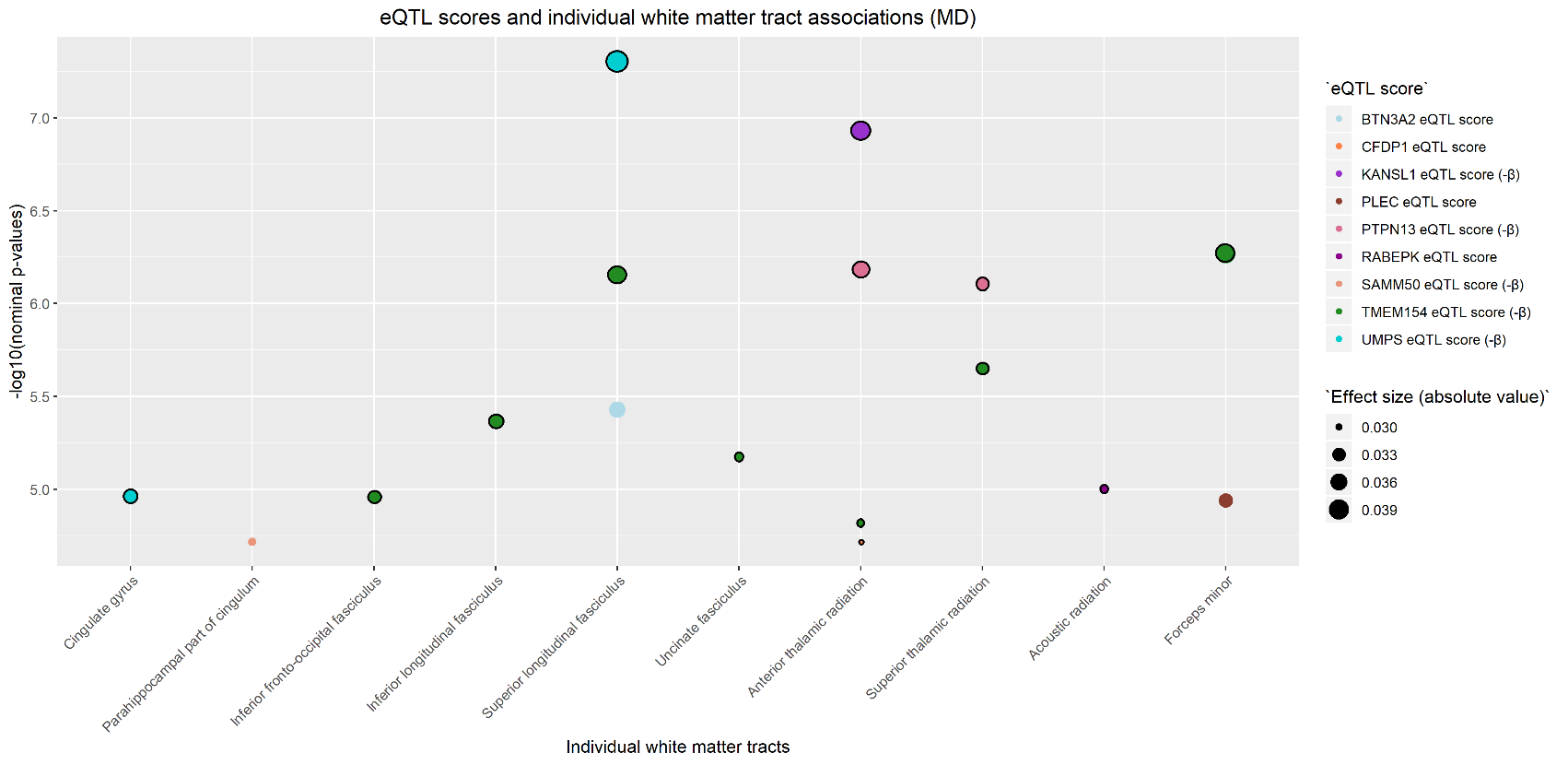

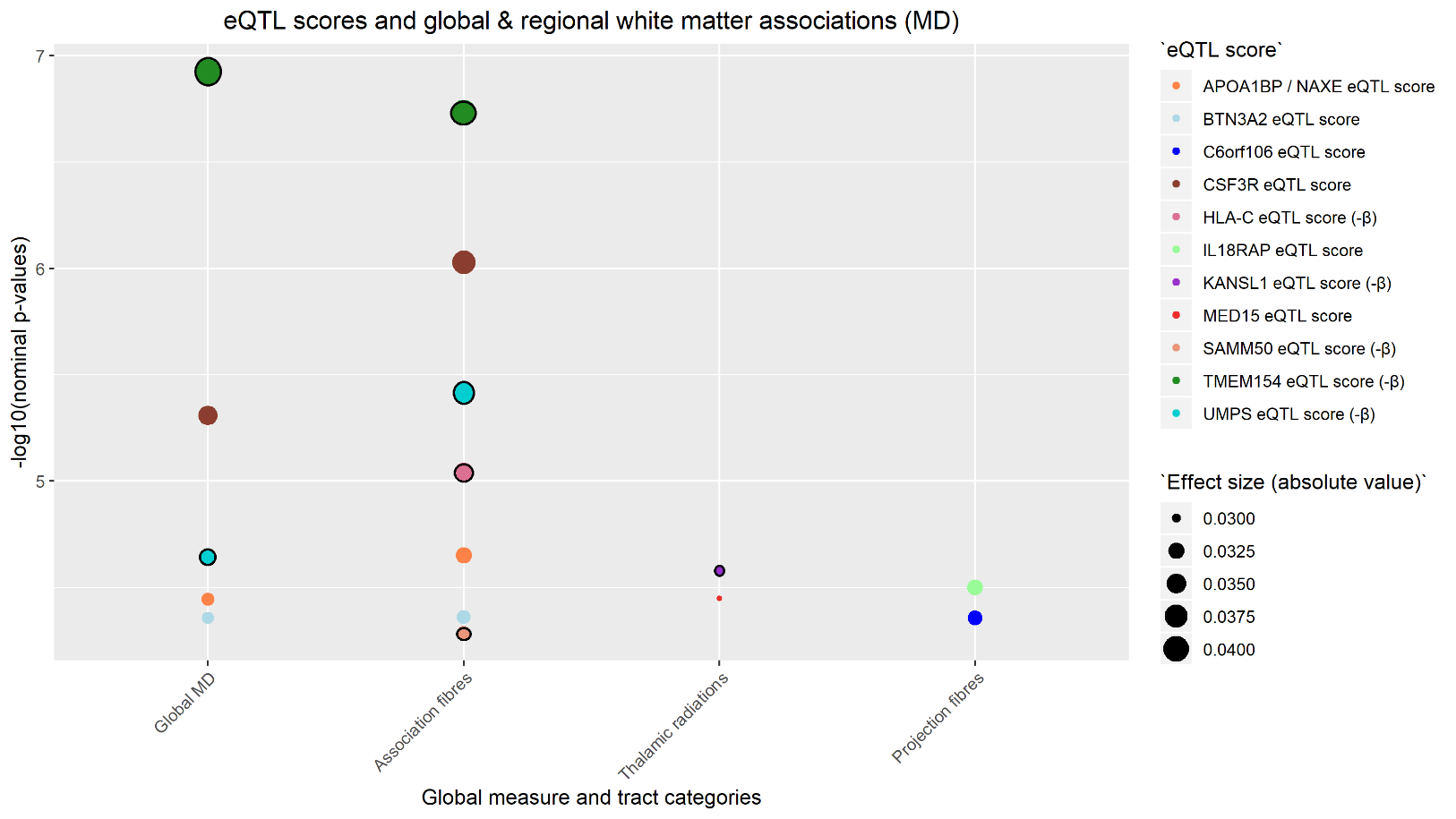
**

**Figure S5.** eQTL scores associated only with white matter tracts as measured by MD (mean diffusivity). Indicates nominal p-values between each of the scores (shown in legend entitled “eQTL score”) and global and tract category measures (noted on the x-axis). All values in the figure met FDR correction. Some of the scores with an additional line around the points had an effect size in the opposite direction to all other scores (also indicated by -β for MD in figure legend). The colours of the plot points indicate the score to which they belong. Magnitude of effect is shown in the legend entitled “Effect size (absolute value)”.

**
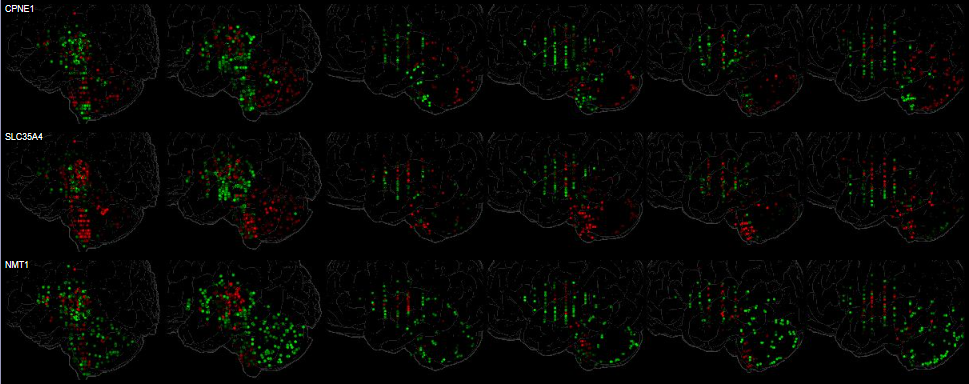

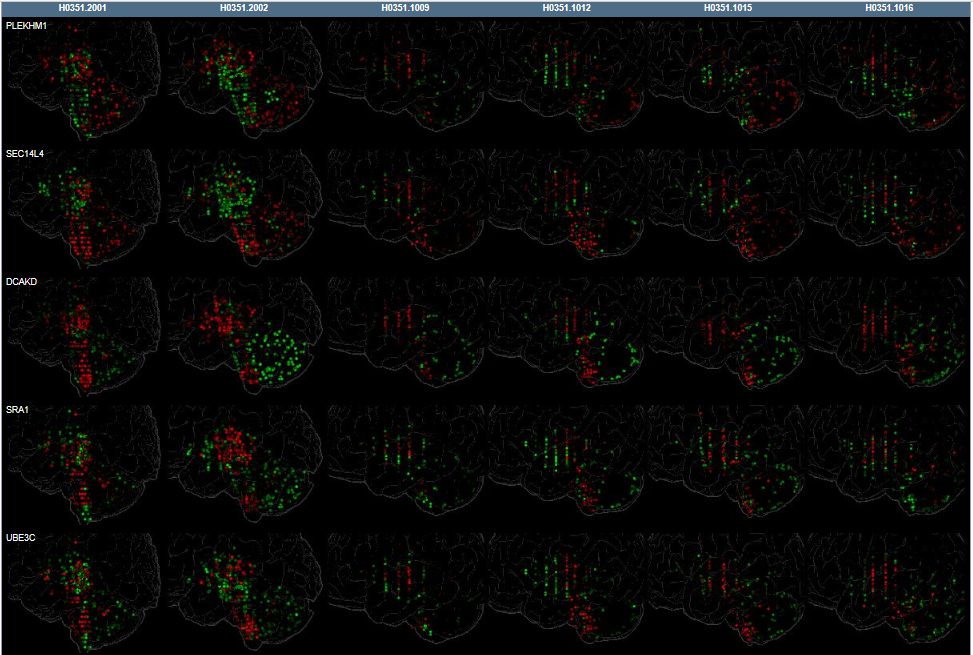
**


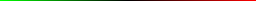



Gene expression scale [-3, 3]

**Figure S6.** Subcortical gene expression patterns for genes regulated by the top 8 eQTL scores. Demographic details about the 6 donors to the Allen Brain Atlas are provided in Table 1. Source: Allen Brain Atlas (<https://portal.brain-map.org/>).


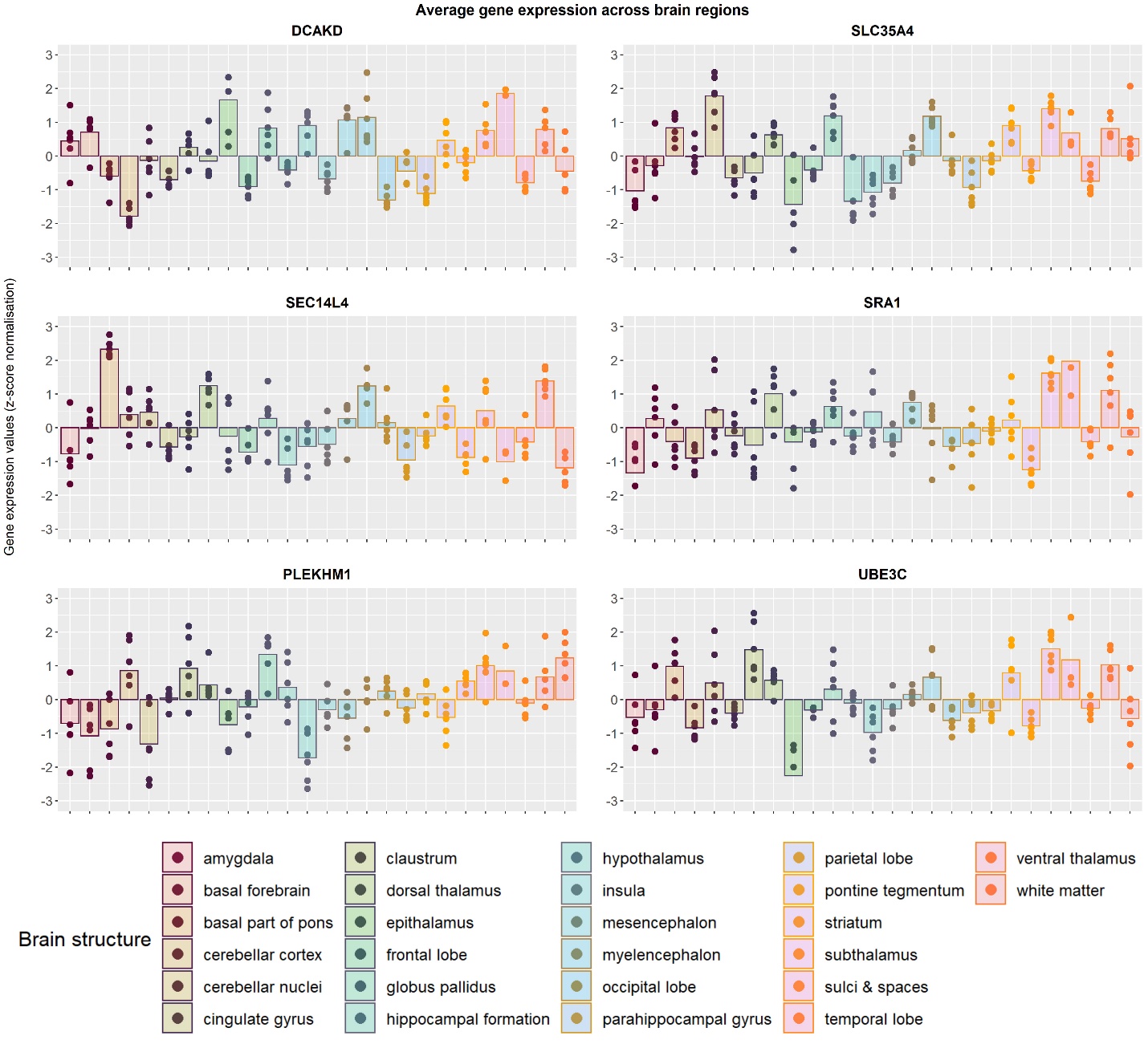


**Figure S7**. Average gene expression patterns across brain regions in N = 6 donors for the 6 disease-linked genes. Points on the plot indicate participants’ own gene expression level, while the bars indicate the mean gene expression value across all donors. The y-axis indicates gene expression values (normalized z-scores). Brain structures are indicated in the legend.

**
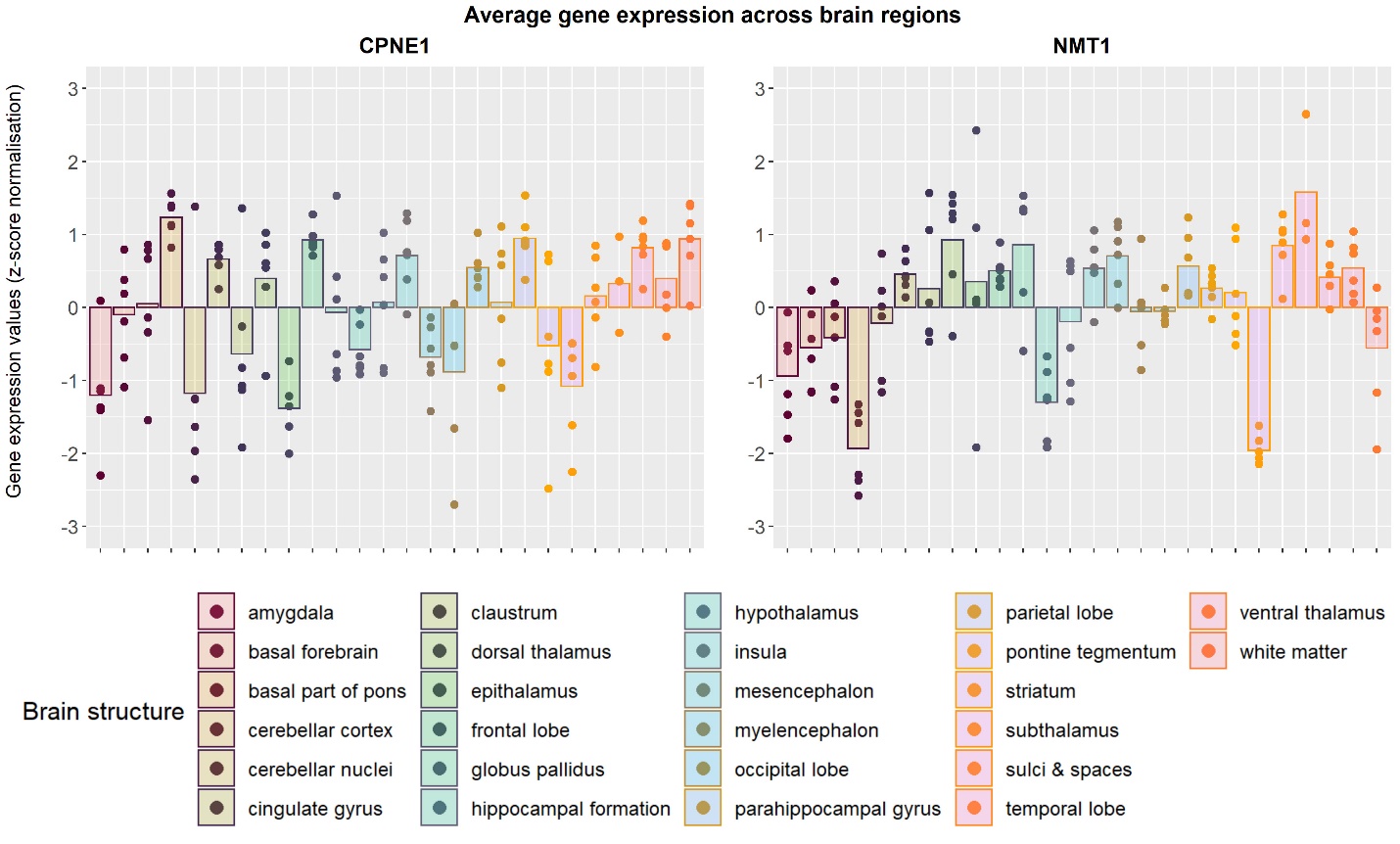
**

**Figure S8**. Average gene expression patterns across brain regions in N = 6 donors for the 2 neurodevelopment-linked genes. Points on the plot indicate participants’ own gene expression level, while the bars indicate the mean gene expression value across all donors. The y-axis indicates gene expression values (normalized z-scores). Brain structures are indicated in the legend.


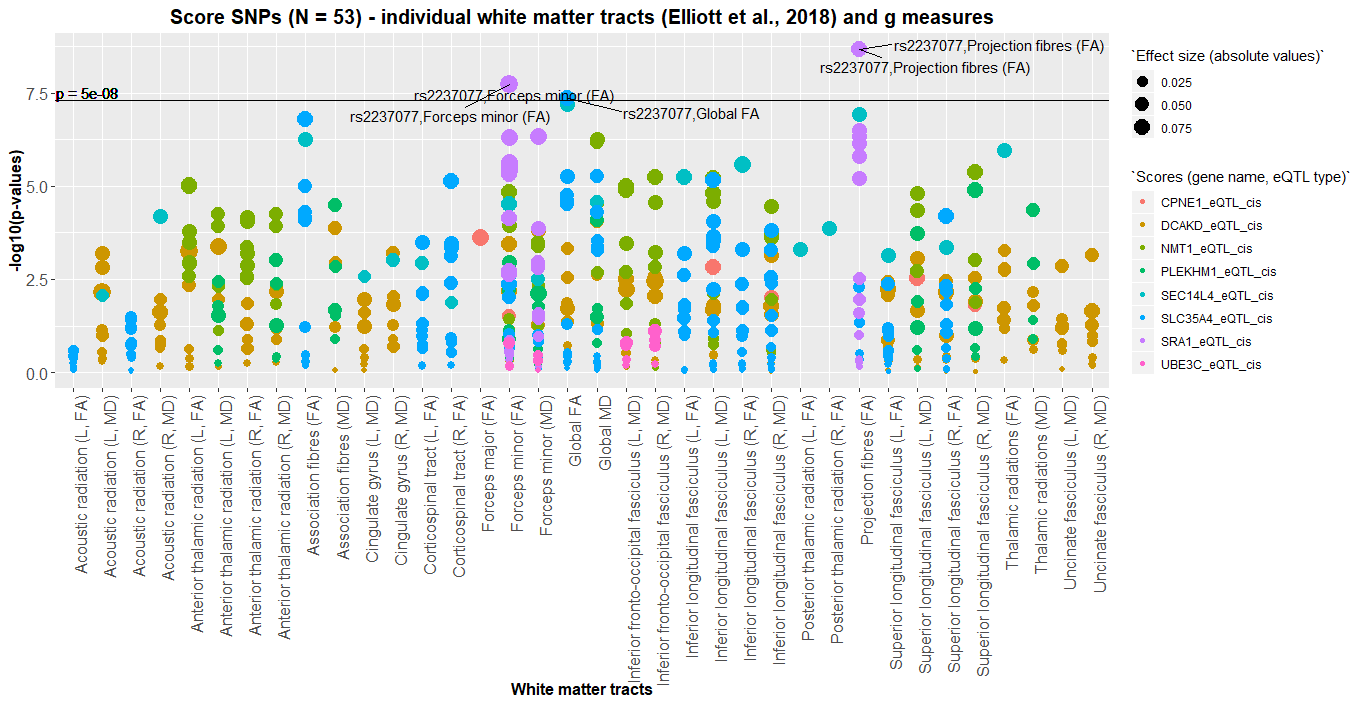


**Figure S9.** GWA between SNPs, individual white matter tracts of interest (Elliott et al., 2018) and global and tract category measures (run locally). Each point on the plot corresponds to one SNP. White matter tracts of interest are noted on the x-axis (L = left; R = right; FA = fractional anisotropy; MD = mean diffusivity). The 8 colours of the plot points indicate the score to which they belong (shown in “Scores (gene name, eQTL type)” legend). Magnitude of effect is shown in the legend entitled “Effect size (absolute values)”. The horizontal line indicates genome-wide significance (5e-8).
